# Cement production and CO_2_ emission cycles in the USA: evidence from MS-ARDL and MS-VARDL causality methods with century-long data

**DOI:** 10.1007/s11356-024-33489-2

**Published:** 2024-05-10

**Authors:** Melike E. Bildirici, Özgür Ömer Ersin

**Affiliations:** 1 Department of Economics, Faculty of Economics and Administrative Sciences, Davutpaşa Campus, Yıldız Technical University, 34220 Istanbul, Turkey; 2 Dept. of International Trade, Faculty of Business, Sütlüce Campus, İstanbul Ticaret University, 34445 Istanbul, Turkey

**Keywords:** Cement, Air pollution, Environmental sustainability, Markov-switching, Cointegration, ARDL, Causality, Q56, L61, B23, C49

## Abstract

The cement industry is among the top three polluters among all industries and the examination of the nonlinear and cointegration dynamics between cement production and CO_2_ emissions has not been explored. Focusing on this research gap, the study employs a novel Markov-switching autoregressive distributed lag (MS-ARDL) model and its generalization to vector error correction, the MS-VARDL model, for regime-dependent causality testing. The new method allows the determination of nonlinear long-run and short-run relations, regime duration, and cement-induced-CO_2_ emission cycles in the USA for a historically long dataset covering 1900–2021. Empirical findings point to nonlinearity in all series and nonlinear cointegration between cement production and cement-induced CO_2_ emissions. The phases of regimes coincide closely with NBER’s official economic cycles for the USA. The second regime, characterized by expansions, lasts twice as long relative to the first, the contractionary regime, which contains severe economic recessions, as well as economic crises, the 1929 Great Depression, the 1973 Oil Crisis, the 2009 Great Recession, and the COVID-19 Shutdown and Wars, including WWI and II. In both regimes, the adverse effects of cement production on CO_2_ emissions cannot be rejected with varying degrees both in the long and the short run. Markov regime-switching vector autoregressive distributed lag (MS-VARDL) causality tests confirm unidirectional causality from cement production to CO_2_ emissions in both regimes. The traditional Granger causality test produces an over-acceptance of causality in a discussed set of cases. Industry-level policy recommendations include investments to help with the shift to green kiln technologies and energy efficiency. National-level policies on renewable energy and carbon capture are also vital considering the energy consumption of cement production.

**Graphical Abstract:**

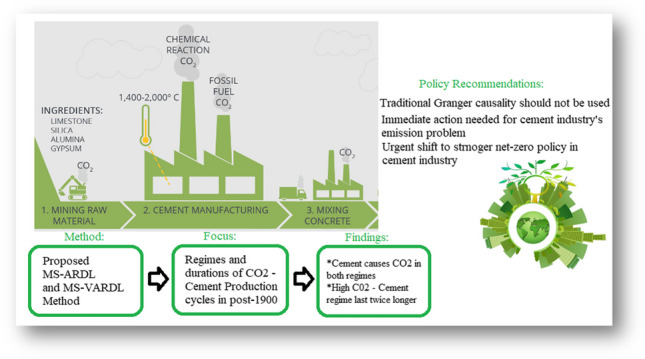

## Introduction

The issue of environmental pollution has significant effects on global warming. The achievement of sustainability in economic development cannot be achieved without environmental sustainability. As a greenhouse gas, carbon dioxide (CO_2_) is among the most important sources of global warming and climate change. In the last century, CO_2_ emissions have risen to unprecedented levels on which industrial production has strong effects. Compared to the pre-industrial levels (1850–1900), the mean temperatures on Earth have been 1.53 °C higher in the last decades and global warming is affecting life on globe through shifts of climate zones, extreme weather events, alterations in the functioning, and structure of climate including the carbon-cycle feedbacks of Earth (Alkama & Cescatti 2016; Forzieri et al. 2017; Hoffman et al. 2014; Richardson et al. 2013). Consequently, if human-induced climate change is not controlled, climate change becomes irreversible. Recent reports from the International Energy Agency (IEA) projects that CO_2_ emissions will further reach a peak of 37 billion tons (Gt) in 2025 (IEA 2022a) and if serious action is not taken soon, global warming will reach an irreversible level in the next 75 years (IEA 2022c) leading to UN Climate Change Report noted the seriousness of the issue and emphasized the insufficiency in the political commitment already existent (UNCC 2022). The report noted that if current policies were to be maintained in the future, CO_2_ emissions would reach a 10.6% increase in 2030, just in 8 years; however, to reverse global warming, the opposite, a cut of 45% is necessary before year 2030 (UNCC 2022).

If all industries in the world are ranked according to the amount of CO_2_ emissions they yield, the cement industry is the top third (The Guardian 2019). If the cement industry were a country itself, it would be the third top CO_2_ emitter after China and the USA. Worrell et al. stressed that cement production was responsible for 5% of the anthropogenic CO_2_ emissions in the early 2000s (Worrell et al. 2001). In 2020, its contribution to global CO_2_ emissions reached 8–10% (Wu et al. 2022). Following these concerns, in the UN’s COP24 meeting that took place in Poland in 2018, cement’s CO_2_ emissions were taken into the goals to revert climate change, with a target of 16% reduction in cement production-induced CO_2_ emissions by 2030 (Rodgers 2018). As a result, the cement sector is one of the most important contributors to CO_2_ emissions in the globe with direct and indirect channels.

The direct channel of the cement-induced CO_2_ emissions is due to the emissions released during the processes in production. There are three main sources of anthropogenic emissions of CO_2_, i.e., fossil fuel oxidation, land-use change (including deforestation), and decomposition of carbonates, and cement production is considered an important emitter mainly in terms of the third (Andrew 2018). CO_2_ is emitted from the calcination process of limestone and the combustion of fuels in the kiln during cement production (Costa and Ribeiro 2020). Reducing the CO_2_ from cement production processes is of great importance. CO_2_ emissions are also released due to the utilization of high levels of energy during production. The CO_2_ statistics stated in the previous paragraph for cement production avoid indirect releases due to the high levels of energy consumed. As shown by Worrell et al. (2001) the total amount of CO_2_ emissions from processing cement and from the energy it necessitates and the average intensity of CO_2_ emissions from global cement production is 222 kg per ton of cement produced. Nagi and Jang stress that the amount is four times higher for Portland cement; each ton of cement produced releases an equal amount of CO_2_ emissions (Naqi & Jang 2019). The CO_2_ emissions of cement accelerate as the share of fossil fuel or nonrenewable energy consumption in the total energy mix is not low and depending on the country and the energy policy followed, the CO_2_ mitigation effect would be altered. In the context of Industry 4.0, the nonrenewable energy consumption share of the USA in its total energy use is shown to be one of the highest (M. Bildirici & Ersin 2023).^1^ As a result, the rigorous commitment to green energy and a large share of renewable energy in the energy mix would help in the reduction of CO_2_ emissions in addition to energy-efficient cement production technologies. As shown in the discussion section, the major cement industries are in China, India, and the USA, and these countries are also among the top countries with high shares of fossil-fuel energy in their energy mixes. As a result, cement industries have not only a national level but also a global effect on CO_2_ emissions.

In addition to its role in environmental pollution, cement is an essential product closely linked to economic development policies and various sectors. Economic development projects are generally coupled with construction projects. The nexus between cement production and economic growth has significant connections to business cycles in the economy; cement production is also subject to interconnected fluctuations in the economy and to the influence of fluctuations in the GDP. Business cycles, which include expansionary and contractionary phases, govern economic activity and construction investments which rise during periods of economic development and growth, which fuel cement production.

The business cycle in the USA is shown to be subject to nonlinearity with the expansionary and recessionary periods with asymmetry in characteristics and durations (Hamilton 1989). These cycles are frequently influenced by economic crises and deep recessions which also bring about fiscal and monetary interventions of the policymakers to bring the economy back to the track of economic growth. It is clear that the economic policy interventions that favor economic expansions had significant and nonlinear effects on environmental sustainability. The production patterns for cement are expected to be highly nonlinear possessing a cyclical tendency that is also connected to economic activity, not to mention, important historical events such as World Wars or deep recessions. Cement production directly emits CO_2_ emissions as a characteristic of cement production and kiln that requires reaching a heat level of 1200 °C. Cement is responsible for 8% of global CO_2_ emissions. In addition to its direct effects, the production requires excessive use of energy that further contributes to CO_2_ emissions. Indirect effects include the CO_2_ emissions geared by construction. Therefore, it is of crucial importance to examine cement production and CO_2_ emissions historically by putting forth the cement-induced CO_2_ emission cycles and their relation to economic cycles in the USA. In addition to these effects, CO_2_ emissions resulting from cement production are expected to be nonlinear and have asymmetric effects that differ in size under distinct regimes with different durations.

With this motivation, the investigation of nonlinear long-run relations and nonlinear causality among cement production and CO_2_ emissions will provide vital information regarding the environmental effects of the cement industry from an empirical perspective. For this purpose, the study employs a long sample starting from 1900 to provide a historical perspective. The sample covers economic contractions, deep crises, and abrupt changes caused by World War I and II, the Great Depression of 1929, the Oil Crisis of 1973, the 2009 Great Recession, and 2020 COVID-19. Therefore, the sample provides a laboratory to examine the CO_2_ and cement production nexus and the cement-induced CO_2_ cycles. The overlook is that these cycles have a relation with economic recessions in the USA; however, the type of recession has a strong influence. As shown in the empirical and discussion sections, cement production and cement-induced CO_2_ emissions fluctuate sharply with deep recessions as well as economic crises and abrupt shocks exampled above. The cycles in the cement-induced CO_2_ emissions and cement production are in close synchronization with the economic business cycles. Given the size of the cement sector among all sectors, its strong influence on the overall CO_2_ emissions of the USA could not be rejected. In addition to the relation of the cement industry with economic cycles in the USA, the relation is not constant, or is linear. The type of the recession matters. We argue that the sector is not affected by short-lasting economic recessions, but has strong relations with longer-lasting and deep recessions, crises, and abrupt changes. The influence of cement on CO_2_ emissions differs in size under expansionary and contractionary cement production regimes. Further, cement manufacture is strongly encouraged by the policymakers during periods of recessions and crises for recovery, in addition to economic expansion periods, to contribute to economic growth or to achieve back its track. As a result, contraction in cement production is not a common situation for all recessions and crises, depending on the type of recession. In many cases, especially for long periods of deep recessions or periods of contractions geared by wars, cement is an important sector with inclines in cement production, which also yields cement-induced CO_2_ emissions.

In light of the discussion above, the goal of the study is to design a nonlinear method to examine the long-run relation between cement production and environmental pollution in the USA with historically long data covering 1900–2021. The reason for choosing the USA is its significant cement production. In fact, in 2015, the cement industry in the USA yielded 82.8 million tons (81,500,000 long tons; 91,300,000 short tons) of cement, valued at US$9.8 billion (DATIS 2020). The USA was ranked as the world’s third-largest cement producer in 2019, trailing behind China and India (USGS 2024). By the end of 2022, cement production in the USA had reached around 95 kilometric tons, placing the nation as the fourth-largest cement producer globally after China, India, and Vietnam^2^ (WPR 2024). On the other hand, there is no long-term data available for China and India, the top two countries for cement production, and the econometric methods employed in this study require data over a long period.

The study suggests a novel approach, the Markov-switching autoregressive distributed lag (MS-ARDL) model by integrating two seminal methods. The MS-ARDL allows modeling regime dynamics and business-cycle modeling benefiting from the dynamic Markov-switching regressions (MSR) of Hamilton (Hamilton 1989). The MS-ARDL approach merges the linear ARDL approach for bound testing and cointegration modeling (Pesaran et al. 2001) with the MSR to obtain a unique approach that captures regime-dependent cointegrated long-run relations and short-run relations with different error correction dynamics to the long-run equilibrium under each regime. The MS-ARDL follows single-step modeling of long- and short-run dynamics similar to the ARDL (Pesaran et al. 2001), which generalizes the well-known two-stage long-run cointegration methodology (Engle & Granger 1987). The proposed model is further generalized to vector autoregressive (VAR) models to obtain the MS-VARDL model in this study. Both MS-ARDL and MS-VARDL models provide insightful information concerning regime durations, cycle dating, and regime-dependent Granger causality investigation for the cement production and cement-induced-CO_2_ emission relation. The contribution of this study to the literature is twofold. Firstly, the study proposes the MS-ARDL and MS-VARDL models, which are expected to provide significant contributions to the empirical analyses, especially in energy and environmental research. Secondly, the contribution of the article to the environmental literature is emphasized by analyzing 123 years of data, highlighting the impact of long-term data usage in this literature.

The paper is structured as follows. The literature review is given in the “Literature review” section, where a discussion of cement-CO_2_ emission relation is evaluated. The econometric methodology for the MS-ARDL model is given in the “Econometric methodology” section. The empirical results are given in the “Econometric results” section. The discussion, policy recommendations, and conclusion are given in the “Conclusion” section.

## Literature review

If the literature on industrial production and emissions is investigated, a large body of research focuses on the positive effects of production on emissions at low levels of production and the relation being reversed at high industrial production levels, the so-called environmental Kuznets curve (EKC). Further, we noted that the empirical literature on the cement and emissions nexus is very limited, especially concerning econometric findings at the national level. The existing recent research focuses mainly on China, and as of our literature search, only a few papers discuss the relation of cement industry emissions in the context of other countries, especially the USA.

The empirical research on emission-gross domestic product (GDP) has gained significant pace following the seminal findings (Grossman & Krueger 1991; Selden & Song 1994; Stern 1994). Grossman and Krueger’s empirical results related the levels of two main pollutants by signifying an inverted-*U*-shaped relation, i.e., environmental pollution increasing (decreasing) at low (high) levels of per capita (Grossman & Krueger 1991), and Selden and Song underlined declining hazardous emissions at high levels of economic development (Selden & Song 1994). The cause of the decline in emissions at high GDP levels was considered as decentralization of industrial production, and the reversal of the positive trend in population growth at high-income levels (Stern 1994). To overcome the impossibility of a negative effect of industrial production on emissions, Lopez recommends internalization of emissions and feedback effects at the industry level and emissions should be taken as a factor of production (Lopez 1994). Convergence of carbon emissions at high GDP levels is an important factor and several empirical findings stressed sigma, stochastic, and beta convergence in addition to the existence of the environmental Kuznets curve (EKC) (Anjum et al. 2014; Pettersson et al. 2014). The existence of a decline in emissions at high industrial production levels is rejected empirically after omitting the bias caused by beta convergence on the empirical methods (Stern et al. 2017).

The long-run and causal effects between energy consumption, growth, and CO_2_ emissions also found significant applications and the importance of energy efficiency and renewable energies were documented (Ozturk & Acaravci 2013). Our findings indicated the close relations of these factors to the cycles in the production of cement; however, these relations are strongly nonlinear both in the short and in the long run, and in addition, our findings suggest the advocation of energy efficiency and green energy policies in the cement industry, which has strong ties with the business cycles of economic growth with differentiated dynamics in the expansionary and recessionary regimes. By investigating the environmental and health effects of the construction industry within a comparative perspective with various sectors, the negative effects of cement production on health and air quality are documented (Bildirici 2020).

By following nonlinear regime switching neural network models and by calculating the sensitivity of CO_2_ growth rates to fossil fuel and economic growth, Bildirici and Ersin emphasize the questionability of linear in parameter-type EKC formulations, in addition to stressing the role of transfer of industrial production to newly industrializing other countries from already industrialized nations (M. Bildirici & Ersin 2018a). Bildirici and Ersin suggest a novel nonlinear STARDL cointegration model, with which important deviations from the EKC are obtained compared to linear ARDL, and it is suggested that CO_2_ and economic growth have nonlinear characteristics due to business cycles, crises, and structural changes in production historically for 1800–2014 period in the USA (M. Bildirici & Ersin 2018b). Using a panel of countries including the OECD countries with the nonlinear Panel STAR model, the EKC relation is strongly rejected in both regimes for the panel of countries (Ersin 2016). Ersin stresses that the turning point threshold is determined by CO_2_ emission growth rates, not the economic growth rates; after the turning point, evidence is against the reversal from environmental degradation under nonlinearity and threshold effects (Ersin 2016).

The consensus in the literature that focuses on the cement industry and its impacts on the environment relates emissions to energy levels needed in production and a common policy recommendation is to increase energy efficiency. However, concerns were also raised about how energy efficiency would slow down the emissions of the cement industry. Accordingly, clinker production activity is identified as the central polluter in the industry (Wang et al. 2013), and estimates show that the cement industry is the highest emitter industry both in China and in the world (Teller et al. 2000). Empirical findings determine labor productivity and energy intensity as major determinants of CO_2_ emissions in the cement sector (Lin & Zhang 2016). Ke et al. confirm the carbon emissions due to the energy intensity of cement production and advocate energy efficiency to lessen emissions (Ke et al. 2012). Xu et al. (2012) distinguish among four features of cement manufacture, overall output, ratio of clinker, processing technique, and type of energy used up per kiln type (J. H. Xu et al. 2012). They identify the link between growth in cement production and economic growth coupled with infrastructure and construction sectors (J. H. Xu et al. 2012).

Specific investigation of the cement industry and its effects on emissions has gained increasing attention and led to important findings (Bekun et al. 2022; Cai et al. 2016; Gao et al. 2017; Ren et al. 2023; Supino et al. 2016; Tan et al. 2022), Further, the majority of empirical research on national data is centered on China with few exceptions. Various studies are investigated which focus on different sectors and among these, some have ties with the cement industry. Regarding important mitigating effects concerning emissions, the emphasized sectors in the literature, other than cement, include petrochemical (Xin et al. 2022), mining (Chen & Yan 2022; Li et al. 2023), logistics (Liang et al. 2022; B. Xu & Xu 2022), transportation (M. Liu et al. 2021; H. Xu et al. 2022), steel and nonferrous metal (J. Zhang et al. 2023), foundry (Zheng et al. 2022), manufacturing industry chains (Lin & Teng 2022), and coal industry (Xia & Zhang 2022). In addition, the construction sector, as a sector related directly to cement consumption, also is among the strong emitters of CO_2_ emissions (Y. Liu et al. 2022; Zhao et al. 2022). The construction sector is followed by sectors of steel, nonferrous sectors, and a fraction of chemical industries as sectors with relations to cement consumption. Concerning the effects of mining (Chen & Yan 2022; Li et al. 2023), the main findings advocate carbon-neutrality policies in the sector to reduce high levels of emissions (Chen & Yan 2022). The nonferrous metal and steel sectors are directly related sectors to the construction sector and have relations to cement consumption. The nonferrous sector has strong effects on CO_2_ mitigation and emission reduction strategies are presented (Cao et al. 2022; Y. Zhang et al. 2022).

Production techniques are criticized in terms of their environmental impacts and alternate techniques are advocated. As an example, the replacement of clinker as the binding material in cement production with recycled material is suggested (Costa and Ribeiro 2020).^3^ Martins et al. study the emissions due to solid, construction, and demolition wastes in addition to the energy consumption created through construction contributing to climate change (Martins et al. 2023). Karlsson et al. calculate a potential 40% reduction in construction-embodied CO_2_ by realizing material efficiency, recycling, and construction supply chains (Karlsson et al. 2021). Though zero-emission is advocated through transforming transportation to electric vehicles (EV), if the very large share of fossil-fuel energy in total energy consumption is taken into consideration, as a typical, over 80% in the USA such an EV policy would have little effect without transformation of energy production from nonrenewables which also has strong emission potential in the installment and maintenance (M. Bildirici & Ersin 2023).

Studies investigated cement production and emissions in selected countries. Hanle et. al is among the few studies, which emphasize the USA’s cement production highlighting the level of CO_2_ emissions it generates (Hanle et al. 2004).^4^ The Dutch construction industry is emphasized in terms of recycled concrete materials to achieve circular economy objectives (Yu et al. 2021). Pakdel et al. investigate the energy-induced CO_2_ mitigation effects of traditional and contemporary methods in the Iranian construction industry (Pakdel et al. 2021). Karlsson et al. explore the Swedish road construction industry through the role of supply chains to achieve net-zero CO_2_ (Karlsson et al. 2020). Huang et al. empirically analyze the nexus between emissions and energy embodied in the construction of buildings in Taipei (Huang et al. 2019). Vorayos and Jaitiang (2020) analyze the relationship between the environment and energy performance of Thailand’s cement industry (Vorayos & Jaitiang 2020). Oke et al. (2017) investigate carbon emission trading in the construction industry in South Africa (Oke et al. 2017). The regional dataset for 2000–2005 and data envelopment techniques for India are used to determine the state-level inefficiency levels of the cement sector and the consequences of CO_2_ emissions (Kumar Mandal & Madheswaran 2010). Turkey’s cement industry is investigated with data envelopment for 51 cement factories in 2016 and CO_2_ emission externality in the cement production process is highlighted (Dirik et al. 2019). These empirical results revealed that only 16% of all integrated cement factories were efficient leading to inclined environmental worsening (Dirik et al. 2019). Belbute and Pereira utilize time-series models with fractional integration to obtain CO_2_ emission forecasts from fossil-fuel consumption and cement production in Portugal and their findings could be interpreted as showing the importance of lowering cement production in achieving carbon emission targets (Belbute & Pereira 2020). By providing a comparative analysis of China and the USA’s cement industry with nonlinear models and Granger causality among cement production, economic growth, and environmental pollution, Bildirici (2019) stresses that if nonlinear relations are ignored, policy recommendations would lead to incorrect results which hamper environmental sustainability (M. E. Bildirici 2019). It is also shown that the effects of cement production and its effects on environmental degradation and health would be insufficiently identified (M. E. Bildirici 2020).^5^

## Econometric methodology

### Single-regime ARDL approach

Cointegration is a seminal technique that allows the researcher to model long-run and short-run dynamics, adjustment towards the long-run equilibrium following shocks, and the length of adjustment in linear relations (Engle & Granger 1987). The ARDL method of Pesaran-Shin-Smith (PSS) generalizes the cointegration method to ARDL methodology with generated testing method of bound tests (Pesaran et al. 2001). The paper aims to generalize the linear, i.e., single-regime, ARDL approach to Markov-switching (MS) to account for nonlinear dynamics in long-run relations.

A single-regime long-run linear regression form is

1 $${Y}_{t}={\delta }_{0}+{\delta }_{1}{X}_{1,t}+{\delta }_{2}{X}_{2,t}+...+{\delta }_{k}{X}_{k,t}+{\eta }_{t}$$

assuming *Y*_*t*_ dependent variable being modeled with *k* number of *X*_*1,t*_,…, *X*_*k,t*_ independent variables, and the long-run form consisting of *k* + 1 number of parameters including the intercept $\left\{{\delta }_{0},{\delta }_{1},{\delta }_{2},...,{\delta }_{k}\right\}$. PSS also allows exogenous variables such as a linear trend, or dummy variable, $D_{t}$, to be included in the long-run form:

2 $${Y}_{t}={\delta }_{0}+{\delta }_{1}{X}_{1,t}+{\delta }_{2}{X}_{2,t}+...+{\delta }_{k}{X}_{k,t}+\varphi {D}_{t}+{\eta }_{t}$$

In the Engle-Granger methodology, the variables have a long-run relation if integrated of a common order *d* and if their linear combination is stationary so that the residuals are stationary (Engle & Granger 1987). In the PSS methodology, the ARDL model allows the combination of *I*(1) and *I*(0) variables; however, to eliminate degenerate cases and loss in power of the ARDL cointegration test, the dependent variable should be *I*(1). The short-run model in which the error-correction presentation is embedded is achieved as

3 $$\Delta {Y}_{t}={\alpha }_{0}+\omega {\eta }_{t-1}+\sum_{i=1}^{m}{\alpha }_{1,i}\Delta {Y}_{t-i}+\sum_{i=1}^{n}{\alpha }_{2,i}\Delta {X}_{1,t-i}+...+\sum_{i=1}^{l}{\alpha }_{k+1,i}\Delta {X}_{k,t-i}+{\varepsilon }_{t}$$

where $\omega$ is the error correction parameter and the speed of transition to the long-run equilibrium is $1/\omega$; for the mechanism to work, it necessitates an estimate of $\omega$ such that $-1<\widehat{\omega }<0$ similar to the Engle-Granger cointegration model (Engle & Granger 1987). For simplicity, in Eq. (3), $\left\{{Y}_{t}, \, {X}_{1,t}, \, \dots ,{X}_{k,t}\right\}\sim I\left(1\right)$, so that ${\Delta }^{d}=\Delta$. However, the Engle-Granger approach is a two-step model in nature given Eqs. (1) and (3). The ARDL model of PSS allows long-run and short-run dynamics to be modeled simultaneously within a single-step estimation,

4 $$\Delta {Y}_{t}={\alpha }_{0}+{\lambda }_{1}{Y}_{t-1}+{\lambda }_{2}{X}_{1,t-1}+...+{\lambda }_{k+1}{X}_{k,t-1}+\varphi {D}_{t}+\sum_{i=1}^{m}{\alpha }_{1,i}\Delta {Y}_{t-i}+\sum_{i=1}^{n}{\alpha }_{2,i}\Delta {X}_{1,t-i}+...+\sum_{i=1}^{l}{\alpha }_{k+1,i}\Delta {X}_{k,t-i}+{\varepsilon }_{t}$$

and further, in Eq. (4), the integration properties of variables are defined as in Pesaran et al. (2001) so that the series is allowed to be *I*(1) or *I*(0) processes or a combination of both (Pesaran et al. 2001). The bound test statistic of Pesaran et. al. (2001), *F*_*PSS*_, is calculated by restricting ${\lambda }_{1}$, ${\lambda }_{2}$,…, ${\lambda }_{k+1}$  = 0 under ${H}_{0}:{\lambda }_{1}=0,{\lambda }_{2}=0,...,{\lambda }_{k+1}=0$, i.e., no cointegration, to be tested against ${H}_{1}:{\lambda }_{1}\ne 0,{\lambda }_{2}\ne 0,...,{\lambda }_{k+1}\ne 0$. If *F*_*PSS*_ > *F*_*PSS,Upper*_ and *F*_*PSS*_ > *F*_*PSS,Lower*_, Pesaran et al. (2001)’s upper and lower bounds, the result would favor cointegration and long-run association (Pesaran et al. 2001). However, confirmation of the existence of a single cointegration vector is necessary (Narayan 2014). The error-correction form is a restricted form as

5 $$\Delta {Y}_{t}={\alpha }_{0}+\omega {\eta }_{t-1}+\varphi {D}_{t}+\sum_{i=1}^{m}{\alpha }_{1,i}\Delta {Y}_{t-i}+\sum_{i=1}^{n}{\alpha }_{2,i}\Delta {X}_{1,t-i}+...+\sum_{i=1}^{l}{\alpha }_{k+1,i}\Delta {X}_{k,t-i}+{\varepsilon }_{t}$$

where $\omega {\eta }_{t-1}$ defines the error-correction mechanism and the previous definitions of ${\eta }_{t}$ and $\omega$ hold. For simplicity, assume *k* = 1. Single-regime ARDL model of Eq. (4) becomes

6 $$\Delta {Y}_{t}={\alpha }_{0}+{\lambda }_{1}{Y}_{t-1}+{\lambda }_{2}{X}_{t-1}+\varphi {D}_{t}+\sum_{i=1}^{m}{\alpha }_{i}\Delta {Y}_{t-i}+\sum_{i=1}^{n}{\beta }_{i}\Delta {X}_{t-i}+{\varepsilon }_{t}$$

and the restricted ARDL representation in Eq. (5):

7 $$\Delta {Y}_{t}={\alpha }_{0}+\omega {\eta }_{t-1}+\varphi {D}_{t}+\sum_{i=1}^{m}{\alpha }_{i}\Delta {Y}_{t-i}+\sum_{i=1}^{n}{\beta }_{i}\Delta {X}_{t-i}+{\varepsilon }_{t}$$

In Eq. (6), single-regime and linear ARDL-type cointegration test hypotheses are ${H}_{0}:{\lambda }_{1}=0,{\lambda }_{2}=0$, ${H}_{1}:{\lambda }_{1}\ne 0,{\lambda }_{2}\ne 0$ and if statistically *F*_*PSS*_ > *F*_*PSS,Upper*_ and *F*_*PSS*_ > *F*_*PSS,Lower*_, the long-run linear association is accepted. If cointegration is established, a confirmatory test is ${H}_{0}:\omega =0$ and ${H}_{1}:\omega \ne 0$ in Eq. (7); the former suggests no linear cointegration, by assuming only a linear form of a long-run association. The above-mentioned ARDL methodology has been challenged and criticized for various aspects: (i) Over-acceptance of cointegration, Narayan’s critical values should be preferred (Narayan 2014), especially for small samples. Further confirmation of the existence of a single cointegration vector is necessary (Narayan 2014). (ii) PSS requires dependent variable to follow ${Y}_{t}\sim I\left(1\right)$ to avoid power loss in the test procedure and to avoid degenerate case-1 (McNown et al. 2018). Under such cases, the *F* or *t-tests* of ARDL cointegration become inconclusive (McNown et al. 2018).^6^ (iii) Bildirici and Ersin (2018a, b) noted ignoring nonlinearity would lead to incorrect policy recommendations and introduce smooth transition ARDL (STARDL) models, by generalizing the single-regime ARDL to smooth transition autoregression (STAR) type nonlinear processes to nonlinear cointegration.^7^

(iv) Banerjee et al. (2017) show the loss of power of the ARDL test under structural breaks and integrate Fourier terms into the ARDL model. Bildirici and Ersin (2023) generalize the proposed Fourier ARDL model to bootstrapping ARDL model to achieve Panel Fourier BARDL to control inefficiency under structural change and nonlinearity (M. Bildirici & Ersin 2023). Banerjee et al. (2017) argue that the Fourier functions with different dimensions could capture various forms and numbers of nonlinear structural changesMetin girmek için buraya tıklayın veya dokunun.. Enders and Lee (2012) show that Fourier is more efficient in correcting the bias in unit root tests under smooth changes and less efficient in abrupt changes (Enders & Lee 2012). MS-type regime models are capable of capturing sudden and abrupt shifts in regimes in addition to determining the dating and duration of regimes.^8^

### Markov regime-switching ARDL model

The MS-ARDL model is a nonlinear error correction (NEC) model that allows nonlinearity in both long-run and short-run dynamics simultaneously. Therefore, MS-type regime changes (Hamilton 1989) are integrated into the ARDL model to achieve the Markov regime-switching autoregressive distributed lag (MS-ARDL) model. Various other forms of NEC are evaluated by Saikkonen (2008). Among these models, Saikkonen (2005) allows regime changes governed by an indicator function to achieve a threshold NEC. Saikkonen (2008) also discusses possible extensions to Markovian regimes to achieve NEC models.

Significant models on modeling NEC have been proposed with various nonlinear techniques. A general tendency for NEC modeling so far has been to keep the short-run parameters linear while allowing error correction parameters to be regime-specific generalizations of Engle-Granger methodology (Kapetanios et al. 2006; Saikkonen 2005, 2008). Krolzig develops the MS-VEC model in a VAR setting and the MS-VEC allows MS-type changes in the error correction as a nonlinear generalization to Engle-Granger’s cointegration approach (H. M. Krolzig et al. 2002).^9^ Pavlyuk applies MS-ARDL model to traffic forecasting (Pavlyuk 2017); however, the model is an MS-ARX model and does not utilize the AR and DL terms in the spirit of NEC modeling and PSS-type ARDL cointegration.

Other NEC models include Kapetanios et al. which allow exponential smooth transition functions to capture regime-dependent error correction (Kapetanios et al. 2006). Shin et al. developed a nonlinear ARDL (NARDL) framework with a threshold-type nonlinearity instead of MS (Shin et al. 2013). Bildirici and Ersin generalize the ARDL to smooth transition type nonlinearity with the smooth transition ARDL (STARDL) model. The STARDL model generalizes ARDL to nonlinearity and asymmetry both for the long- and short-run relations (M. Bildirici & Ersin 2018b). With this respect, both STARDL and the NARDL models relax the symmetry assumption for either the long- or the short-run terms.

An MS-ARDL model with two or more regimes is

8 $$\Delta {Y}_{t}={\alpha }_{0}^{{s}_{t}}+{\lambda }_{1}^{{s}_{t}}{Y}_{t-1}+{\lambda }_{2}^{{s}_{t}}{X}_{1,t-1}+...+{\lambda }_{k+1}^{{s}_{t}}{X}_{k,t-1}+\sum_{i=1}^{m}{\alpha }_{1,i}^{{s}_{t}}\Delta {Y}_{t-i}+\sum_{i=1}^{n}{\alpha }_{2,i}^{{s}_{t}}\Delta {X}_{1,t-i}+...+\sum_{i=1}^{l}{\alpha }_{k+1,i}^{{s}_{t}}\Delta {X}_{k,t-i}+{\varepsilon }_{t}^{{s}_{t}}$$

where ${\alpha }^{{s}_{t}}={\left\{{\alpha }_{0}^{{s}_{t}},{\alpha }_{1,i}^{{s}_{t}},{\alpha }_{2,i}^{{s}_{t}}...,{\alpha }_{k+1}^{{s}_{t}}\right\}}^{\prime}$ is the short and ${\lambda }^{{s}_{t}}={\left\{{\lambda }_{1}^{{s}_{t}},{\lambda }_{2}^{{s}_{t}},...,{\lambda }_{k+1}^{{s}_{t}}\right\}}^{\prime}$ is the long-run parameter vector, both being regime-dependent; regime changes are governed with *s*_*t*_ for *r* number of regimes ${s}_{t}\in \left\{\mathrm{1,2},...,r\right\}$. Hence, *s*_*t*_ = 1, *s*_*t*_ = 2,…, and *s*_*t*_ = *r* is a finite regime sequence. $N\left( 0,\sum\left( s_{t} \right) \right)$ is distributed with zero conditional mean and regime-dependent $\sum \left({s}_{t}\right)$ nonnegative conditional variance. As a result, ${\varepsilon }_{t}^{{s}_{t}}$ are allowed to be locally homoskedastic for sub-regression spaces, while being globally heteroskedastic. For a similar approach, see Saikkonen (2008). The model generalizes the single-regime ARDL in Eq. (4) to MS-type regime switches in Eq. (8).

*The generalization of ARDL bound testing* is necessary in the MS-ARDL modeling stages. Once the existence of MS-ARDL type nonlinearity is accepted against linear ARDL following the Davies linearity test, the null hypothesis of no cointegration relation is

9 $$H_0:\lambda_1^{s_t}=\lambda_2^{s_t}=...=\lambda_{k+1}^{s_t}=0\;for\;s_t=1,2,...,r$$

which means neither linear nor nonlinear error correction exists, to be tested against the alternative of MS-type nonlinear cointegration,

10 $$H_1:\lambda_1^{s_t}\neq\lambda_2^{s_t}\neq...\neq\lambda_{k+1}^{s_t}\neq0\;for\;s_t=1,2,...,r$$

defining a regime-dependent cointegration in each distinct regime. The testing requires a conventional *F* test approach, the calculated *F* statistic is *F*_*MSARDL*_, and if it passes both the upper and lower bounds, *F*_*MSARDL*_ > *F*_*PSS,upper*_ and *F*_*MSARDL*_ > *F*_*PSS,Lower*_, the *H*_0_ null hypothesis of no cointegration is rejected against the alternative *H*_1_, that is, MS-ARDL-type cointegration with *r* number of regimes. The proposed *F*_*MSARDL*_ test statistic follows an *F* distribution, *F*(*q*,*n* − *r*(*k* + 1) − 1), with *q* = *r*(*k* + 1) where* r* represents the number of regimes and* k* + 1 is the number of ${\lambda }^{{s}_{t}}$ tested for cointegration for each regime.

By replacing the long-run part with the regime-specific error correction mechanism, reduced form nonlinear MS-ARDL error correction representation of Eq. (8) is

11 $$\Delta {Y}_{t}={\alpha }_{0}^{{s}_{t}}+{\omega }^{{s}_{t}}{\eta }_{t-1}+\sum_{i=1}^{m}{\alpha }_{1,i}^{{s}_{t}}\Delta {Y}_{t-i}+\sum_{i=1}^{n}{\alpha }_{2,i}^{{s}_{t}}\Delta {X}_{1,t-i}+...+\sum_{i=1}^{l}{\alpha }_{k+1,i}^{{s}_{t}}\Delta {X}_{k,t-i}+{\varepsilon }_{t}^{{s}_{t}}$$

${\omega }^{{s}_{t}}$ is a regime-specific error correction parameter for ${\eta }_{t-1}$, the error-correction term. If the error correction parameter estimate, ${\widehat{\omega }}^{{s}_{t}}$, is statistically accepted to lie between $-1<{\widehat{\omega }}^{ {s}_{t}=r}<0$, regime-specific error correction duration is calculated as 1/${\omega }^{{s}_{t}}$, which holds for each *r* distinct regimes as long as ${\widehat{\omega }}^{ {s}_{t}=1}\ne {\widehat{\omega }}^{ {s}_{t}=2}\ne ...\ne {\widehat{\omega }}^{ {s}_{t}=r}$.^10^

The conditional probability density of time series *y*_*t*_ is stated as

12 $$P({y}_{t}{\left|{Y}_{t-1},s\right.}_{t-1})=\left\{\begin{array}{c}f{\left(\left.{y}_{t}\right|{Y}_{t-1},{\phi }_{1}\right)}i{f}{s}_{t-1}=1\\ f{\left(\left.{y}_{t}\right|{Y}_{t-1},{\phi }_{2}\right)}i{f}{s}_{t-1}=2\\{\vdots }\vdots \\ f{\left(\left.{y}_{t}\right|{Y}_{t-1},{\phi }_{r}\right)}i{f}{s}_{t-1}=R\end{array}\right.$$

where ${\phi }_{r}$ is the vector of parameters in *r* = 1,2,*…*,*r* number of regimes (H.-M. Krolzig 1997). The Markov chain defining the regime-switching process for the model is as follows:

13 $${p}_{ij}=P\left[{s}_{t}=i\left|{s}_{t-1}=j\right.\right]=P\left(\left.{s}_{t}\right|{s}_{t-1}\right){,}\sum_{j}^{r}{p}_{ij}={1}$$

where *p*_*ij*_ is the probability of being in regime *i* at time *t* conditional on the state (or regime) *j* at time *t − *1 (Hamilton 1989). Similar to the MS-AR and MS-VAR models, *p*_*ij*_ is subject to

14 $$p_{ij}=P\left(s_t\left|\left\{s_{t-1}\right\}_{i=1}^\infty,\left\{y_{t-1}\right\}_{i=1}^\infty\right.\right)=P\left(\left.s_t\right|s_{t-1};\;\rho\right)$$

where $P\left\{\left.{s}_{t}\right|{s}_{t-1};\rho \right\}$ is the probability of state *s*_*t*_ at period *t* conditional on the previous state *s*_*t − 1*_ (M. E. Bildirici 2020). The switching variable, *s*_*t*_, is an unobserved discrete-state Markov chain, which governs the endogenous switches in *r* number of regimes (Krolzig & Toro 2002).^11^ In each distinct regime, a locally linear ARDL sub-space exists defining regime-specific relations among modeled time series. Hence, it is an irreducible ergodic Markov process with *r* number of states for which the transition matrix is (Hamilton 1989)

15 $$\bf P = \left[ {\begin{array}{cccc} p_{11} & p_{12} & \cdots & p_{1r}\\ p_{21} & p_{22} & \cdots & p_{2r}\\ \vdots & \vdots & \ddots & \vdots\\ p_{r1} & p_{r2} & \cdots & p_{rr}\\ \end{array} } \right]$$

Consistent with the MS-VAR and MS-AR models, the Markov chain follows the irreducible and ergodic process and each *p*_*ij*_ has an unconditional and stationary distribution given the ergodicity of the Markov process (H.-M. Krolzig 1997). The probability of ${s}_{t-1}=i$ at *t − *1 is conditional on the information set available and the parameter set, $\Omega_{t-1};\;\phi_r$. Hence, in the iteration process, for *t* = 1, 2, …, *T*, the probability for the previous period is used as an input:

16 $$\xi_{it-1}=P\left[s_{t-1}=i\left|\Omega_{t-1};\;\phi_r\right.\right]$$

The present state ${\xi }_{it}$ includes all information regarding the Markovian process that follows in the future (H.-M. Krolzig 1997):

17 $$P\left(\left.{\xi }_{it+1}\right|{\xi }_{it},{\xi }_{it-1},{\xi }_{it-2},...,{\xi }_{it-T};{y}_{t},{y}_{t-1},...,{y}_{t-T}\right)=P\left(\left.{\xi }_{it+1}\right|{\xi }_{it}\right)$$

The conditional log-likelihood is stated as $\log\;f\;(y_1,y_2,...,y_T\vert y_0;\;\phi)=\sum\log\;f\;(y_t\vert\Omega_{t-1};\;\phi)$.

For a two-regime MS-ARDL model, Eqs. (15) and (16) become.

18 $$P=\left[\begin{array}{cc}{p}_{11}& {p}_{12}\\ {p}_{21}& {p}_{22}\end{array}\right]$$

19 $$\xi_{it}=P\left[s_t=i\vert\Omega_t;\;\phi\right],\;i=1,2$$

where the unconditional distribution of each *p*_*ij*_ is

20 $${p}_{ij}=P\left[{s}_{t}=i\left|{s}_{t-1}=j,{s}_{t-2}=k,{s}_{t-3}=l,...{s}_{t-T}=z\right.\right]=P\left(\left.{s}_{t}=i\right|{s}_{t-1}=j\right)={p}_{ij}$$

and the calculation leads to

21 $$P\left({s}_{t}=1\right)=\frac{1-{p}_{22}}{2-\left({p}_{11}+{p}_{22}\right)},P\left({s}_{t}=2\right)=\frac{1-{p}_{11}}{2-\left({p}_{11}+{p}_{22}\right)}$$

In the case of two regimes, observations are conveyed into the first sub-regression space if $Pr({s}_{t}= \, 1\left|{Y}_{T}\right.)\ge 0.5$ or to the second if $Pr({s}_{t}= \, 1\left|{Y}_{T}\right.)<0.5$. In the estimation step, the expectation maximization (EM) algorithm is utilized (Hamilton 1989).

### Markov regime-switching vector autoregressive distributed lag model

The MS-ARDL model assumes both the long-run and short-run dynamics to follow nonlinear regime-switching. A vector autoregressive (VAR) generalization of the MS-ARDL model is necessary to investigate the existence of a single cointegration vector. In addition, the MS-VARDL model could be easily adapted to examine nonlinear Granger causality between the analyzed variables depending on the distinct regimes. Therefore, it is convenient to write the Markov-switching vector ARDL (MS-VARDL) model as a VAR generalization of Eq. (8). For simplicity, a two-variable, two-regime MS-VARDL model is given as

22 $$\begin{array}{c}\Delta {Y}_{t}={\alpha }_{0}^{{s}_{t}}+{\lambda }_{1}^{{s}_{t}}{Y}_{t-1}+{\lambda }_{2}^{{s}_{t}}{X}_{1,t-1}+{\varphi }_{1}{D}_{t}+\sum_{i=1}^{m}{\alpha }_{1,i}^{{s}_{t}}\Delta {Y}_{t-i}+\sum_{i=1}^{n}{\alpha }_{2,i}^{{s}_{t}}\Delta {X}_{1,t-i}+{\varepsilon }_{1,t}^{{s}_{t}}\\ \Delta {X}_{t}={\theta }_{0}^{{s}_{t}}+{\tau }_{1}^{{s}_{t}}{X}_{1,t-1}+{\tau }_{2}^{{s}_{t}}{Y}_{t-1}+{\varphi }_{2}{D}_{t}+\sum_{i=1}^{m}{\theta }_{1,i}^{{s}_{t}}\Delta {Y}_{t-i}+\sum_{i=1}^{n}{\theta }_{2,i}^{{s}_{t}}\Delta {X}_{1,t-i}+{\varepsilon }_{2,t}^{{s}_{t}}\end{array}$$

where ${\alpha }_{1,i}^{{s}_{t}},{\alpha }_{2,i}^{{s}_{t}}$ and ${\theta }_{1,i}^{{s}_{t}},{\theta }_{2,i}^{{s}_{t}}$ are the short-run parameter sets in MS-VARDL vectors 1 and 2 and ${\lambda }_{1}^{{s}_{t}},{\lambda }_{2}^{{s}_{t}}$ and ${\tau }_{1}^{{s}_{t}},{\tau }_{2}^{{s}_{t}}$ are the vector-specific long-run parameters. Given that ${s}_{t}=\mathrm{1,2}$, cointegration testing necessitates repeating the MS-ARDL cointegration test separately for each vector in Eq. (22) for ${\lambda }_{1}^{{s}_{t}},{\lambda }_{2}^{{s}_{t}}$ and for $\tau_{1}^{{s_{t} }} ,\tau_{2}^{{s_{t} }}$. In vector 1, zero-restricted $\lambda_{1}^{{s_{t} }} ,\lambda_{2}^{{s_{t} }}$ lead to the null hypothesis of no cointegration (linear or nonlinear) in both regimes:

23 $$H_0:\lambda_1^{s_t}=\lambda_2^{s_t}=0\;for\;s_t=1,2$$

24 $$H_1:\lambda_1^{s_t}\neq\lambda_2^{s_t}\neq0\;for\;s_t=1,2$$

For the second vector, the null of no-cointegration,

25 $$H_0:\tau_1^{s_t}=\tau_2^{s_t}=0\;for\;s_t=1,2$$

is tested against nonlinear cointegration as

26 $$H_1:\tau_1^{s_t}\neq\tau_2^{s_t}\neq0\;for\;s_t=1,2$$

For both tests,* F*_*MSARDL*_ test statistic follows an *F* distribution as $F_{MSARDL} \sim F(q,n - r\left( {m + n + k + 1) - 2} \right)$ and for the two-variate, two-regime model, *q* = 4. As a next step, one could also estimate a restricted error correction form of MS-VARDL for confirmatory purposes:

27 $$\triangle Y_t=\alpha_0^{S_t}+\omega_1^{S_t}\eta_{1,t-1}+\varphi_1D_t+\sum \limits_{i=1}^m\alpha_{1,i}^{S_t}\triangle Y_{t-i}+\sum \limits_{i=1}^n\alpha_{2,i}^{S_t}\triangle X_{1,t-i}+\varepsilon_{1,t}^{S_t} \triangle X_t=\theta_0^{S_t}+\omega_2^{S_t}\eta_{1,t-1}+\varphi_2D_t+\sum \limits_{i=1}^m\theta_{1,i}^{S_t}\triangle Y_{t-i}+\sum \limits_{i=1}^n\theta_{2,i}^{S_t}\triangle X_{1,t-i}+\varepsilon_{2,t}^{S_t}$$

To test no-cointegration (linear or nonlinear) against nonlinear cointegration, hypotheses are $H_{0} :\omega_{1}^{{s_{t} }} = 0$ and ${H}_{1}:{\omega }_{1}^{{s}_{t}}\ne {0}{{\text{for}}}{s}_{t}=\mathrm{1,2}$ in vector 1, and $H_{0} :\omega_{2}^{{s_{t} }} = 0$ and $H_{1} :\omega_{2}^{{s_{t} }} \ne 0$ in vector 2 of the model given in Eq. (27). Vector-specific *F*_*MS-VARDL*_ test statistic follows $F_{MS - VARDL} \sim (2,n - r\left( {m + n + 1} \right) - 2)$. The MS-VARDL modeling steps proposed above aim at testing nonlinear ARDL-type error correction occurring in each vector. For specific applications, researchers could also consider testing the existence of regime-specific nonlinear cointegration (M. Bildirici & Ersin 2018b). In this case, once MS-VARDL given in Eq. (22) is estimated sub-tests targeting specific regimes for specific vectors are likely. As a typical, assume testing regime 1 of vector 1, a low volatility or economic growth regime. Regime-specific hypotheses are $H_{0} :\lambda_{1}^{{s_{t} = 1}} = \lambda_{2}^{{s_{t} = 1}} = 0$, $H_{1} :\lambda_{1}^{{s_{t} = 1}} \ne \lambda_{2}^{{s_{t} = 1}} \ne 0$. For regime-specific or global nonlinear cointegration, readers are referred to M. Bildirici and Ersin (2018b).

To achieve the existence of multiple regimes, Davies tests should be applied. Further, the stability of the ergodic switching probabilities should be examined with the diagonal of Eq. (15) or in a two-regime model, the *p*_11_ and *p*_22_ in Eq. (18) so that *p*_11_ < 0.5, *p*_22_ < 0.5 to achieve persistence in each regime in addition to confirming their statistical significance.

MS-VARDL in Eq. (22) reduces to the MS vector error correction (MS-VEC) model (H.-M. Krolzig & Toro 2002) under very mild restrictions applied on the long- and short-term parameters. The MS-VEC generalizes the VEC model to MS and cointegration methodology. The MS-VARDL, on the other hand, generalizes MS-ARDL to MS-VARDL. The nonlinear MS-VEC model is given as (Clements & Krolzig 2002; H.-M. Krolzig 1997)

28 $$\Delta Y_t-\delta^{S_t}=\alpha(\beta^{\prime}Y_{t-1}-\mu^{S_t}-\varphi D_t)+\sum \limits_{k=1}^{p-1}B_i(\Delta Y_{t-i}-\delta^{S_t})+\varepsilon_t$$

$\delta^{{s_{t} }}$ is a drift term that is a function that shifts the intercept in the long-run equation. $\beta^{\prime}$ is the long-run parameter vector and *B*_i_ is the short-run parameter set. The short-run parameters are not subject to regime-switching. *Y*_*t*_ is the variable matrix and the model is distinguished as a *shifting mean regime-switching model* for *s*_*t*_ = 1,2,…,*r* number of regimes. By applying zero restrictions to an MS-VARDL, the reduced form MS-VEC representation exists.^12^

## Econometric results

The study will focus on the following steps in the empirical section:

1. Unit root (UR) testing with a battery of tests allowing different forms of data-generating processes. Included tests are ADF, KPSS, KSS, and F-ADF. KPSS is robust to various forms of structural breaks, the KSS test (Kapetanios et al. 2006) tests unit root against nonlinear stationary series, and F-ADF is the Fourier ADF test of Engle and Lee (2011) known to be robust to a wide form of nonlinear series in addition to smooth structural breaks.
2. *F* bound testing with traditional single-regime ARDL and Johansen cointegration test to investigate the existence of cointegration.
3. The BDS test (Broock et al. 1996) is applied to investigate the nonlinearity of the series.
4. Nonlinear regime-dependent bound testing is tested with the MS-ARDL test.
5. Single-regime ARDL and nonlinear MS-ARDL models are estimated.
6. Determination of regime durations, datings, and regime switching probabilities for MS-ARDL.
7. Linearity is tested against regime-dependent nonlinearity with *F* tests.
8. Model evaluation with diagnostics tests.
9. The determination of the direction of causality and comparative analysis with single-regime causality (VEC-based) and regime-switching causality (MS-VARDL-based).
10. Inference and policy recommendations following the direction of causality determination.

### Data

The study is among one of the studies that utilize historically long datasets in the literature focusing on environmental sustainability within econometric respects. In terms of the evaluation of the effects of cement on the environment, the study is, to our knowledge, a pioneering study that evaluates a historically long sample for the post-1900 period in terms of focusing on the econometric relations between CO_2_ emissions and cement production.^13^ The sample covers the 1900–2021 period for the USA and the dataset is yearly. The period contains several significant events, including the First and Second World Wars, the 1973 Oil Crises, and important economic crises, such as the 1929 Great Depression, the Great Recession in 2008, and recently, COVID-19. The emission data represents CO_2_ emissions from cement production in the USA and is in kilotons of CO_2_. Cement production (*CP*_*t*_) is in billion metric tons and is available from the Andrew (2022) database which obtains the yearly *CP*_*t*_ data from the U.S. Geological Survey (Andrew 2018, 2022). Variables are subject to natural logarithms as *LCO*_*t*_ = ln(*CO*_*2t*_) and *LCP*_*t*_ = ln(*CP*_*t*_). As reported in the following section, these level series contain unit roots and are integrated of order 1. After first differencing, the respectful series are Δ*LCO*_*t*_ and Δ*LCP*_*t*_, which also represent the growth rates. The descriptive statistics are reported in Table 1.

**Table 1:** Descriptive statistics

	*Mean*	*Max*	*Skew*	*Kur*	*JB*	*p*
*LCO*_*t*_	6.4673	7.7547	0.404049	2.41871	2.394709	0.43
*LCP*_*t*_	5.36063	6.40305	− 0.213185	2.78391	3.71268	0.29

JB is the Jarque–Bera test of normality test statistic and *p* is the probability. *Sd*, *Skew*, and *Kur* represent the standard deviation, skewness, and kurtosis statistics, respectively

For the level series, Jarque–Bera test statistics imply that, at 5% level of significance, normality for level variables cannot be rejected. For the first differenced series, series are not normally distributed and are subject to skewness and excess kurtosis. In the next step, series are tested for stationarity and unit roots.

### Unit root and stationarity tests

The unit root tests are used to determine the order of integration of the series and whether the variables are *I*(0) or *I*(1). A battery of tests are applied which include traditional tests in addition to those robust to various forms of nonlinearity. These tests include the Augmented Dickey-Fuller (ADF), Kwiatkowski-Phillips-Schmidt-Shin (KPSS), Kapetanios-Shin-Snell (KSS), and Enders and Lee (2011) Fourier-ADF. The test results are reported in Table 2. The traditional linear unit root test, mainly the ADF test, is known to have size distortions under nonlinearity and structural changes and the ADF test tends to over-reject the null hypothesis of unit root (Nelson et al. 2001). KPSS tests stationarity under the null and the test is known to be less influenced by nonlinear series. The KSS tests the null of the unit root series against the alternative of stationary time series following a nonlinear STAR process under the alternative. Enders and Lee’s (2012) Fourier ADF test is utilized to test the unit root null hypothesis and the test is known to be robust against smooth forms of structural changes. The unit root tests utilized in the study evaluate stationarity under forms of structural changes and nonlinearity. In all tests reported in Table 2, both *LCP*_*t*_ and *LCO*_*t*_ series are integrated of a common order of 1, and they become stationary once first differenced.

**Table 2:** Unit root and stationarity test results

*Variables*	*ADF*	*KPSS*	*KSS*	*F-ADF*
*LCP*_*t*_	− 2.080	0.902	− 2.1419	− 1.203
*∆CLP*_*t*_	− 8.053***	0.2301***	− 6.6712***	− 6.733***
*LCO*_*t*_	− 2.237	0.894	− 1.5296	− 0.921
*∆LCO*_*t*_	− 10.70***	0.17***	− 4.8225***	− 4.6629**

Δ is the first difference. ***, **, and * denote the significance at *α* = 0.01, 0.05, and 0.10 significance levels. ADF and KPSS tests are calculated for intercept + trend assumption. The critical values for the ADF tests are − 3.486551, − 2.886074, and − 2.579931 at *α* = 0.01, 0.05, and 0.10 significance levels. For the KPSS test, the spectral estimation and bandwidth selection are conducted with Bartlett kernel and Newey-West bandwidth methods for which the critical values are 0.739, 0.463, and 0.347 at *α* = 0.01, 0.05, and 0.10. In the KSS STAR-type unit root test, Cases 1, 2, and 3 (C1, C2, and C3) represent raw, demeaned, and detrended data selections, respectively. Following Table 1 of KSS (2003), the critical values for C3 are − 3.93, − 3.40, and − 3.13 at *α* = 0.01, 0.05, and 0.10 significance levels. F-ADF represents the Fourier-ADF test of Enders and Lee (2012). For Fourier dimension *k* = 1, the critical values of tau test statistic (Table 1 of EL, 2022) are − 4.69, − 4.10, and − 3.82 at *α* = 0.01, 0.05, and 0.10 statistical significance levels

### BDS test results

Broock, Deckert, and Sheinkman (BDS) test is a test based on correlation dimension and the test examines the independent and identically distributed *i.i.d.* series under the null hypothesis against the alternative of nonnormality caused by series following nonlinear or chaotic behavior. Broock et al. (1996) provide a recent treatment of the test (Broock et al. 1996). BDS test provides an investigation of deviations from independence and is known to be efficient in detecting different forms of nonlinearity. BDS test is also suggested as a model architecture determination tool similar to tests such as the Ljung-Box test of autocorrelation; hence, the test could also be used on residuals of models to investigate remaining nonlinearity (Broock et al. 1996).

It should be noted that the unit root and stationarity tests determined that series in levels follow *I*(1) processes and the model in the next section will utilize both the levels and the first differenced series. In Table 3, BDS test results are given for series in levels and for series in first differences. For robustness, the variables are also tested with the Tsay test of threshold-type nonlinearity (Tsay 1986). The results are given in the last column of Table 3.

**Table 3:** BDS and Tsay test results

BDS test					Tsay test		
	*LCO*_*t*_		*LCP*_*t*_			*LCO*_*t*_	*LCP*_*t*_
*D*	*BDS*	*z*	*BDS*	*z*	*l*	*F*	*F*
2	0.19	22.83***	0.15	13.32***	1	2.21*	3.03***
3	0.32	24.08***	0.22	12.23***	2	5.41***	7.53***
4	0.40	25.58***	0.24	11.02***	3	4.62***	5.01***
5	0.47	27.86***	0.23	9.99***			
6	0.51	31.15***	0.19	8.33***			
	*∆LCO*_*t*_		*∆LCP*_*t*_			*∆LCO*_*t*_	*∆LCP*_*t*_
*D*	*BDS*	*z*	*BDS*	*z*	*l*	*F*	*F*
2	0.15	19.12***	0.18	28.96***	1	2.91**	2.35**
3	0.24	19.78***	0.30	30.39***	2	4.20***	3.01**
4	0.29	20.35***	0.39	32.47***	3	4.05***	2.28*
5	0.33	21.56***	0.44	35.35***			
6	0.35	23.26***	0.47	39.25***			

*d* is the embedded dimensions between 2 and 6 (default), *BDS* and *z* are BDS and *z* test statistics. Tsay test *F* statistic is calculated up to *l* = 1,2,3 lags where the optimum lag length is determined as 3 with Akaike information criteria. Significance at 10%, 5%, and 1% significance levels are given with *, **, and ***, respectively

Since BDS and *z*-test statistics are greater than the critical values at conventional significance levels, the null hypothesis of *i.i.d.* is rejected for both *LCO*_*t*_ and *LCP*_*t*_, and results favor nonrejection of the alternative hypothesis suggesting that series in levels follow nonlinear processes. For their first differenced counterparts, BDS results suggest similar results at conventional significance levels for all embedded dimensions the test is conducted for. Tsay test results are given in the last section of Table 3, where the test is repeated for different lag orders. The Tsay test results confirm the rejection of linearity for both level and first differenced series at conventional significance levels against the alternative of nonlinearity. The overall results suggest that linear models could not be appropriate due to the nonlinear characteristics of the data analyzed.

### MS-ARDL dating of contractions in cement production-emission cycle

In the first stage, we examined the estimated MS-ARDL model in terms of its relation with the NBER contraction (including economic recessions and crises) periods. The results are reported in Table 4.

**Table 4:** Comparison of NBER economic contraction dates with model results for the USA

Model results capturing deep recessions and economic crises*	NBER contraction dates for the USA economy**
1907–1909	1908, 1911
1914–1916	1914–1915
1917–1921	1919–1921
-	Last quarter of 1924–last quarter of 1925
1930–1933	Last quarter of 1927–1928
	1933–1935
1938–1939	1938–1939
1945–1946	1945–1946
-	1949 (11 months)
-	1954 (10 months)
-	1958 (8 months)
-	1961 (10 months)
-	1970 (11 months)
1974–1976	Late 1974–mid-1976 (16 months)
1980–1981	1980 (6 months)
	late 1982–mid-1983 (16 months)
1991–1994	1991 (8 months)
-	2001 (8 months)
2008–2011	2009–2010
2020–2021	2020

*Model focuses especially on deep recessions that last more than a year and economic crisis dates due to the nature of the data of cement-production and cement production-induced CO_2_ emissions in the USA. Short recessions (less than a year) with less impacts are omitted since they fail to transform into sharp production declines in the industry’s output

**NBER cycles are obtained from NBER (2023), available from https://www.nber.org/research/data/us-business-cycle-expansions-and-contractions (accessed at 14.1.2023)

The first column includes the estimated contraction dates for cement production in the cement industry and the CO_2_ emissions due to cement production. The second column reports the contraction periods reported by the NBER (National Bureau of Economic Recessions). Therefore, the table provides NBER and the authors’ calculations obtained from the MS-ARDL which will provide important insights. The overall look shows that for the majority, the dates and durations match with those reported by NBER. It should be noted that an exact match should not be expected all the time. NBER dating focuses on economic recessions in the whole industry and the model results focus on cement-industry and cement-induced CO_2_ emissions. Though NBER dates and cement-CO_2_ cycles are generally in line, it is also expected to have leading and especially lagging relations in the CO_2_ emissions instead of exact coincidences all the time. The general outlook is, though these are not in general, they occur mainly after deep recessions and crises and after such periods, once expansion in the economy starts, cement production and especially CO_2_ emissions follow in certain additional years which leads to acceleration in cement-induced CO_2_ cycles. Investigation of a set of selected periods will hinder important information regarding the cement production and CO_2_ emission cycles and their relations to the economy as a whole.

NBER contraction dates are calculated from NBER’s business cycle reference dates. NBER announces trough dates and the duration of contraction periods in months starting from the date of the trough. Though NBER cycles are for the economy as a whole, the contraction dates and durations reported by the MS-ARDL model estimations are for cement-induced CO_2_ emissions and cement production. The findings suggest that, depending on the economic recession, the cycle dates do not match all the time. As a result, the findings suggest that the cement production and CO_2_ cycle generally lags the economy’s business cycle in general except for distinct cases such as the 1914–1916 contraction which shows that the industry was already in crisis. Further, one could combine 1914–1916 and 1917–1921 contractions which totals 1914–1921, which coincides with the 1914–1915 and 1919–1921 economic cycles. It should be noted that these economic cycle dates coincide with WWI and the post-WW1 period explaining the length and duration of the contraction in the cement output and cement-induced CO_2_ emissions. Another cycle starting in 1991 is in line with the year of the Golf War. The economic contraction was estimated to be for 1991–1992 by NBER. With our calculations benefiting from the estimated MS-ARDL model, the recession is estimated for 1991–1994, 2 more years, compared to the NBER cycle for the cement-production and cement-induced CO_2_ emission relation. Findings show that following deep recessions and wars, tightening in cement production and cement-induced CO_2_ emissions follow with a lag. However, it quickly shifts back again to the track of increasing emissions which occurs during the expansionary economic periods. For other dates, we note that the cycles reported by NBER and the MS-ARDL estimations are close matches. The WWII period is taken into consideration as a significant case that lasted between 1939 and 1945. The NBER dates are 1938–1939 and 1945–1946 which present the contraction years which match with those for the cement production and cement-based CO_2_ emission cycle.

Another important finding is that the model catches the dates especially crisis periods and deep recessions especially lasting more than 1 year. Given that the model utilizes cement production instead of cement consumption, these findings are expected. Cement consumption responds quickly through declines in demand in the recession periods. However, cement production continues production and stocks the excess supply as inventories and this is especially so in the short recessions which take between 6 and 11 months. As typical, the 1949 recession lasted 11 months according to NBER but no contractions were captured for cement production and cement-induced CO_2_ emissions. If growth rates are to be noted, cement production declined by 12% in both 1980 and 1982 and for the cement industry, more drastic declines are captured by the MS-ARDL model. For example, cement production declined by 27% in 1918 (WWI) and 39% in 1944 (WWII), but only a 6% decline in 1930, just after the 1929 Great Depression. In 1931, cement production declined by 31% and by 50% in 1932 corresponding to the deep recession period afterward. After the Oil Crisis in 1973–1974, cement production decreased by 18%. Further, the decline amounted to 30% in 2009, the Great Recession, and only 12% weakening in early 2020 during the COVID-19 shutdown.^14^ The findings confirm that cement production responds to deep recessions and crises and during mild recessions, the effects of business-cycle contractions are relatively less on the cement industry and therefore on cement production-induced CO_2_ emissions in such periods. This is expected since both variables are production-based and during recessions and expansions, cement consumption responds relatively fast and with moderate response to economic fluctuations. Consequently, cement-production-induced CO_2_ emissions react to profound recessions and economic crises by deteriorating CO_2_ emissions. Otherwise, the emitting effect continues given the level of cement production under mild recessions.

### Linear (single-regime) ARDL results

To provide a baseline analysis, linear, single-regime ARDL modeling steps are conducted. Results are reported in Table 5. Investigation of cointegration with the ARDL model also provides crucial input for the determination of the direction of cointegration in addition to providing the opportunity to investigate the residuals for remaining nonlinearity which cannot be captured with the linear model.

**Table 5:** Pesaran ARDL bound and Johansen cointegration tests

*Architecture*	*F*_*PSS*_	*p*	
*ARDL model: LCO*_*t*_ = *f*(*LCP*_*t*_)			
ARDL(1,1)	13.745***	0.001	
*ARDL model: LCP*_*t*_ = *f*(*LCO*_*t*_)			
ARDL(1,1)	3.1081	0.2502	
*Johansen cointegration test, variables: LCO*_*t*_*, LCP*_*t*_			
No. of C.E	Trace	5% C.V	*p*
None	19.55689**	15.49471	0.03
At most 1	1.853215	3.841466	0.17

Model architecture is selected with Schwarz information criterion (SC). AIC is the Akaike’s information criterion. LL is the log-likelihood. *F*_*PSS*_ is the Pesaran et al. (2021) ARDL bound test statistic and *p* is the probability of accepting *H*_0_ of no ARDL-type cointegration. In the Johansen cointegration test, null of no cointegration equations at rank *r* is tested. Trace is the trace test statistic and MacKinnon-Haug-Michelis *p* values are reported

The ARDL model is determined as ARDL(1,1) with Schwarz information criterion (SC). By taking each variable as the dependent variable one by one, bound tests are repeated. *F*_*PSS*_ is calculated as 13.745, favoring cointegration, if *LCO*_*t*_ is assumed as the dependent variable, the cement-production-induced CO_2_. For the counter case, by taking *LCP*_*t*_ as the dependent, *F*_*PSS*_ = 3.1081, at the 1% significance level, *F*_*PSS*_ < *F*_*lower*_ and* F*_*PSS*_ < *F*_*upper*_, leading to the decision of no cointegration. (Narayan 2014; Pesaran et al. 2001). The results favored cointegration relation and long-run association between *LCO*_*t*_ and *LCP*_*t*_ and the direction of relation is determined as *LCO*_*t*_ = *f*(*LCP*_*t*_). For confirmatory analysis, the Johansen cointegration test is conducted. Results are reported in the second part of Table 5. Johansen test confirms a single cointegration equation for the *LCO*_*t*_ and *LCP*_*t*_ relation.

The selected ARDL model is tested with the BDS test (Broock et al. 1996) and results are reported in Table 6. As suggested by Broock et al. (1996), the BDS test could be used as a test of remaining nonlinearity if used for the residuals of the model. Accordingly, the linear ARDL model fails to capture the nonlinearity in the residuals and therefore the nonlinear relation between cement production and CO_2_ emissions at conventional significance levels. Hence, the linear ARDL model cannot be accepted under remaining nonlinearity and to avoid possible inefficient policy recommendations, nonlinear ARDL methods should be followed (M. Bildirici & Ersin 2018b). The analysis will continue with nonlinear MS-ARDL-type nonlinearity testing and modeling.

**Table 6:** Remaining nonlinearity test results

*Dim*	*BDS*	*se*	*z*	*p*
2	0.038924	0.010254	3.795933	0.0001
3	0.080927	0.016433	4.924556	0.0000
4	0.103766	0.019743	5.255899	0.0000
5	0.118840	0.020765	5.722998	0.0000
6	0.121325	0.020212	6.002705	0.0000

*Dim* is the embedded dimension in the BDS test. *BDS*, *z*, *se*, and *p* are the BDS statistic, *z* statistic, standard error, and *p* value

### MS-ARDL nonlinear cointegration test results

In this step, the null of linearity in residuals of the linear ARDL model is tested against the alternative of MS-ARDL-type nonlinearity. The Davies-type linearity test statistic is calculated as *F* = 18.0521, favoring the rejection of the single-regime model against the regime-switching model with 2 regimes. After that, the MS-ARDL test of no cointegration is conducted. In the test, no cointegration (linear or nonlinear) is tested under the null hypothesis against nonlinear cointegration. The test results are given in Table 7.

**Table 7:** MS-ARDL bound test results

*Architecture*	*F*_*PSS*_	*p*
*MS-ARDL model: LCO*_*t*_ = *f*(*LCP*_*t*_)		
*MS(2)-ARDL(2,2)*	9.882477***	0.00
*ARDL model: LCP*_*t*_ = *f*(*LCO*_*t*_)		
*MS(2)-ARDL(2,2)*	3.98456	0.17

Model architecture is selected SC. *F*_*MS-ARDL*_ test tests *H*_0_ of no linear or nonlinear cointegration against the alternative of nonlinear cointegration. Narayan (2004) critical values are utilized for *p* value calculation

Assuming *LCO*_*t*_ as the dependent variable, *F*_*MS-ARDL*_ test statistic = 9.882477, statistically larger than the 5% critical lower and upper bounds and the results show that *LCO*_*t*_ and *LCP*_*t*_ have a nonlinear relation in the long run. By assuming *LCP*_*t*_ as the dependent variable, a second *F*_*MS-ARDL*_ is calculated and is equal to 3.98456, lower than the upper and lower critical *F* values, leading to the rejection of cointegration at conventional significance levels. Hence, the results determined nonlinear ARDL cointegration between cement production and cement-production-induced CO_2_ emissions in addition to determining a single cointegration vector exists only by taking the emissions as the dependent variable.^15^

### Model estimation results

Following the nonlinear cointegration test results, a two-regime MS-ARDL model is estimated. In addition, the linear single-regime ARDL model is estimated for comparative purposes. Following Tables 6 and 7, cement-induced CO_2_ emissions are taken as the dependent variable for both the ARDL and MS-ARDL models, and optimum lag length is selected as three for the ARDL and as two for the MS-ARDL model with the Schwarz information criterion. The MS-ARDL model is determined to have two regimes following the linearity tests.^16^ The ARDL and MS-ARDL model estimation results are given in Table 8 where the long-run dynamics are reported in the first part followed by the short-run dynamics, the regime-switching probabilities, and the diagnostic test results.

**Table 8:** MS-ARDL model estimation results

Model:	ARDL	MS-ARDL	
Variable:	Linear	Regime 1	Regime 2
***Long-run dynamics***			
*LCO*_*t − 1*_	0.897179*** [8.08]	− 0.061569*** [− 2.80906]	− 0.1256** [− 1.9038]
*LCO*_*t* − *2*_	− 0.167068 [1.49]	− 0.021402*** [− 2.80074]	0.231* [1.67184]
*LCP*_*t* − *1*_	1.542830*** [5.606]	0.286435** [1.97321]	0.1669*** [2.66417]
*LCP*_*t* − *2*_	− 1.336262*** [5.25]	0.198493* [1.75217]	0.159*** [2.60208]
*cons*	− 0.360971 [0.49]	− 0.007557 [− 0.54908]	0.040408 [0.47065]
***Short-run dynamics***			
Δ*LCO*_*t* − *1*_	0.727153 [1.485]	− 0.10289** [− 2.40968]	− 0.00137** [− 2.1154]
Δ*LCO*_*t* − *-2*_	1.382953* [1.836]	− 0.09644** [− 2.31488]	− 0.0456** [− 2.38168]
Δ*LCP*_*t* − *1*_	− 0.845787* [1.775]	− 0.60017** [− 2.36082]	− 0.0007** [− 2.03254]
Δ*LCP*_*t* − *2*_	− 0.445492* [1.698]	0.790901** [2.50498]	0.14275** [2.19862]
*cons*	1.389129 [1.113]	0.221451** [2.40422]	0.013853* [1.86914]
*ECT*_*t* − *1*_	− 0.01502 [1.3964]	− 0.25748*** [− 18.5004]	− 0.48296*** [− 4.64320]
*Sigma*	-	0.1801***	0.0969***
***Regime-switching probabilities***			
***p_{1|1}***	-	0.88500***	
***p_{2|2}***	-	0.940845***	
***Diagnostics***			
*R*^*2*^	0.74761	0.856924	
*Adj.R*^*2*^	0.641777	0.842617	
*LL*	94.89146	32.22177	
*AIC*	− 3.004436	− 17.30127	
*SC*	− 2.787434	− 13.49496	
*F*_*RESET*_	3.015793	1.20535	
*BPG*_*LM*_	9.095007	1.31172	

*t* test statistics are reported in brackets after each parameter estimate. *, **, and *** denote the statistical significance at 10%, 5%, and 1% levels of statistical significance, respectively. *LL* is the log-likelihood, *AIC* and *SC* are the Akaike and Schwarz information criteria, *F* Reset is the *F* test statistic for Ramsey's Reset test of model misspecification,* BPG LM* is the Lagrange multiplier test statistic for the Breusch-Pagan-Godfrey test of homoskedasticity

The linear ARDL model estimation results are in column 1. Once evaluated, though the parameters of cement production are significant in the long run suggesting the net effect of cement production to be positive on emissions, certain results lead us to be cautious about the ARDL model results. First, the error correction mechanism fails to hold since the parameter of *ECT*_*t −* 1_ is estimated as − 0.015, suggesting an error correction duration approaching 66 years, relatively too long compared to the nonlinear and regime-specific error correction parameter estimates; however, the results are not reliable for the linear model due to the factors listed below. Additionally, the error correction term is statistically insignificant for the linear model, suggesting that the error correction cannot be established for the linear model. The remainder of the linear ARDL model is evaluated with BDS and RESET tests. The BDS test results in Table 6 confirmed the remaining nonlinearity in the residuals of the linear ARDL, possibly leading to biased parameter estimates if nonlinearity in the series is ignored. Among the diagnostics tests, Breusch-Pagan-Godfrey (BPG) and Ramsey’s RESET tests reported in the last section favored homoskedastic residuals, but the model was mis-specified at a 5% significance level. If evaluated together, the estimation results of the linear ARDL model fail to produce satisfactory results, and due to remaining nonlinearity in the residuals, the ARDL parameters for the linear model, if interpreted and if taken for policy purposes, would be seriously misleading.

If the diagnostics tests are evaluated for the MS-ARDL model, the goodness of fit statistics of the MS-ARDL model favors a better fit of the nonlinear model over its single-regime counterpart. Ramsey’s RESET and Breusch-Pagan-Godfrey (BPG) test results for the linear model conclude model-misspecification and heteroskedastic residuals at conventional statistical significance levels. The BPG and RESET test results for the MS-ARDL model favor no homoskedasticity in the residuals and no-model-misspecification. The BPG and RESET tests favor no misspecification and homoscedastic residuals for the MS-ARDL model. Once considered together with the nonlinearity test results, diagnostics tests lead to the existence of remaining nonlinearity in the residuals in the ARDL model and selection for the MS-ARDL results over its linear counterpart. For the MS-ARDL model results reported in columns 2 and 3, the remaining nonlinearity in the residuals is tested with the BDS test, and test results confirm no remaining nonlinearity. As a result, the estimation results of the MS-ARDL model will be taken as central in the study to examine the relations between cement production and emissions.

For the MS-ARDL results given in Table 8, the first and second regimes correspond to the recession and crisis regime (regime 1) and expansion regime (regime 2), respectively. If an overlook is presented to the estimated nonlinear long-run dynamics, it is striking that the impact of cement production on CO_2_ emissions is positive in both regimes with different magnitudes. In the long-run equation of regime 1, the relevant coefficients of *LCP*_*t − 1*_ and *LCP*_*t − 2*_ are estimated as 0.286 and 0.198, and in regime 2, 0.167 and 0.159, respectively. The parameters are statistically significant at the 5% significance level, with one exception: the parameter of *LCP*_*t − 2*_ is significant at the 10% significance level only. Hence, a 1% increase in cement production at periods *t − *1 and *t − *2 leads to 0.286% and 0.198% increases in the CO_2_ emissions in regime 1, compared to 0.167% and 0.159% effects in regime 2, the last one being significant at 10% only, but the positive effect persists. The overall result is that the positive effects of cement production on CO_2_ emissions cannot be rejected in the USA for the long run. It should be noted that the positive effect of cement production is positive in both regimes.

The short-run dynamics are presented in the second section of Table 8. Once the parameters of Δ*LCP*_*t*_ are investigated for regimes, they confirm the effects of cement production on CO_2_ emissions in the short-run in addition to the long-run dynamics presented. Similarly, the findings confirm regime-dependent and asymmetric effects of cement production in the short run. The parameter estimates for Δ*LCP*_*t − 1*_ and Δ*LCP*_*t − 2*_ are − 0.60 and 0.79 in regime 1 and − 0.0007 and 0.14275 in regime 2. Though a 1% increase in the previous year’s cement production decreased emissions by 0.60 in regime 1, the same parameter in regime 2 is estimated as − 0.0007, and almost no effect exists in regime 2 for the first lag of Δ*LCP*_*t* − *1*_. However, for the second lag, the parameter estimate of Δ*LCP*_*t* − *2*_ is estimated as 0.14, significant at 5% significance level, and a 1% increase leading to a 0.79% increase in emissions in regime 1 and 0.14% increase in regime 2. Short-run dynamics also confirm significant and positive impacts of cement production on emissions during both regimes and in terms of the long-run portion of the model, this positive effect increases especially during the cement production expansion regimes.

The significance, sign, and size of the error correction terms play a crucial role in establishing cointegration. The error correction parameters are estimated as − 0.25748 and − 0.48296 for regimes 1 and 2. Hence, 25.7% (48.3%) of the deviations from the long-run equilibrium are corrected within 1 period, and the error correction towards the long-run equilibrium takes 3.9 (2.07) years in regime 1 (regime 2). Overall results show that asymmetry and regime dependence are important factors determining the effects of cement production on emissions. The effects of cement production are positive in both regimes while being larger both in the short and in the long run. Additionally, the error correction mechanisms are asymmetric among regimes; the mechanism takes twice as long in regime 1 compared to regime 2. The estimated probability of staying at the regime at period *t* conditional on the regime at period *t* − 1 determines the persistence of both regimes. The estimated regime probabilities are *p*(*s*_*t*_ = 1 | *s*_*t*_ = 1) = 0.885 and *p*(*s*_*t*_ = 2 | *s*_*t*_ = 2) = 0.940845 for regimes 1 and 2, signifying a high degree of persistence in both regimes and regime 2 being relatively more persistent and longer lasting.

### MS-ARDL causality results

In the next section, MS-ARDL-based causality analysis is reported. The determination of the direction of causality under regime dependency plays a crucial role in policy suggestions. In the context of the methodology presented in the “Econometric methodology” section, once the MS-ARDL model is extended to the MS-VARDL model, regime-specific Granger noncausality test results are calculated and reported in Table 9. For comparative purposes, linear noncausality test results are reported in the last column.

**Table 9:** Comparative linear and nonlinear causality tests

	*MS-VARDL-based regime-specific causality*		*VAR-based linear Granger causality*
*H*_*0*_*: no Granger causality*	*Regime 1*	*Regime 2*	*Linear (single-regime)*
Δ*LCP*_*t*_ $\nrightarrow$ Δ*LCO*_*t*_	2.360919**	2.183221**	2.05263**
*Result:*	*H*_*0*_* rejected*	*H*_*0*_* rejected*	*H*_*0*_* rejected*
Δ*LCO*_*t*_ $\nrightarrow$ Δ*LCP*_*t*_	1.064823	2.032117**	0.18013
*Result:*	*H*_*0*_* accepted*	*H*_*0*_* rejected*	*H*_*0*_* accepted*
*Direction of causality*	*Unidirectional* Δ*LCP*_*t*_ → Δ*LCO*_*t*_	*Bidirectional* Δ*LCP*_*t*_ → Δ*LCO*_*t*_ Δ*LCO*_*t*_ → Δ*LCP*_*t*_	*Unidirectional* Δ*LCP*_*t*_ → Δ*LCO*_*t*_
*Sign of causal effect*	+	+ , +	+

*t* test statistics are reported. *, **, and *** denote the statistical significance at 10, 5, and 1%. $\nrightarrow$ means no causality, → shows the direction of causality. *Sign of causal effect* is based on the statistical significance of relevant parameter estimates at 5% significance level

The regime-specific causality results could be easily determined through the utilization of the methods followed in the paper. According to our results, the null hypothesis of Granger noncausality from cement production to CO_2_ is rejected and the alternative is accepted in both regimes in addition to the linear model given in the last section. Hence, the results confirm nonlinear unidirectional causality from cement production to emissions in regime 1, the high-emission regime. This finding is in line with the linear causality test results. However, by investigating regime-specific causality results, our model provides bidirectional causality between cement production and CO_2_ in regime 2. As a result, the method the paper provides led to additional insights given the feedback effects specific to regime 2. Hence, the feedback effect due to bidirectional causality is a phenomenon occurring specifically in regime 2, the low-emission regime, in contrast to the unidirectional causal effect from cement production to emissions in regime 1.

Given the fact that the nonlinear causality testing utilizes the MS-VARDL results, after the determination of the directions of causality, our method also allows the determination of the sign of the causal effect by evaluating the regime-specific parameter estimates.^17^ The results are given in the last row of Table 9. At the 5% significance level, for the determined causal effects in both regimes, the signs of parameters are positive confirming the positive effects of cement production on emissions in all specifications in addition to the positive effect of emissions on the acceleration of cement production in regime 2.

The findings of this paper indicate that reducing CO_2_ emissions is contingent upon curtailing cement production, as it is the primary source of CO_2_ emissions and this result is obtained in both regimes. Consequently, though asymmetry between the effects of cement production on CO_2_ emissions exists, this asymmetry is mainly in terms of the magnitude, but not in terms of the sign of the effect. In addition, nonlinear causality results provided important deviations from the traditional causality results obtained with linear Granger causality techniques. If the findings are evaluated as a whole, the MS-ARDL results are led to the same results as the regime-dependent causality results and confirm these results. Traditional causality results are in line with the causality results in regime 1. Conversely, the causality results in changes in regime 2. Thus, the policy suggestions should be determined independently for regimes 1 and 2, the deep recession and crisis regime, and the expansionary regime, respectively.

Following the estimation results above, interesting results are obtained once the linear ARDL and nonlinear MS-ARDL are considered. The general finding suggests that regime dependency and nonlinearity should be taken into consideration for policy suggestions focusing on the negative effects of the cement industry on environmentally hazardous greenhouse gases. Depending on the regime, such negative effects accelerate and policymakers should consider the regime the economy and the industry are in since the CO_2_ emitting effect of the industry depends on the regime type. Utilizing the outcomes of MS-VARDL analysis for nonlinear causality assessment, our approach not only establishes the causal directions but also enables the determination of the causal effect’s polarity through an examination of regime-specific parameter estimations. These outcomes are presented in the final row of Table 9. At a significance level of 5%, the parameter signs for established causal effects in both regimes are positive, affirming the beneficial impact of cement production on emissions across all specifications. Furthermore, a positive influence of emissions on the acceleration of cement production is confirmed in regime 2. When considering the entirety of the findings, the MS-ARDL results align with regime-dependent causality outcomes, thereby reinforcing these conclusions. Traditional causality results are consistent with the causality outcomes observed in regime 1. In contrast, the causality directions diverge in regime 2. Accordingly, causality results affirm nonlinear unidirectional causality from cement production to emissions within regime 1. In terms of unidirectional links, the findings align with the findings of the linear causality tests for regime 1 only. The nonlinear method reveals bidirectional causality between cement production and CO_2_ emissions in regime 2, in contrast to the unidirectional causality in regime 1. As a result, the method presented in this paper yields supplementary insights by uncovering feedback effects specific to regime 2, and the regimes that the industry is at gives vital information since the feedback effect could lead to a circular effect resulting in a cycle of more emissions. Hence, the phenomenon of feedback effects stemming from bidirectional causality manifests uniquely within regime 2, in contrast to the unidirectional causal effect from cement production to emissions observed in regime 1, and policies should aim at avoiding the negative implications on the environment by aiming at the alteration of the type of industrial production techniques with newer technologies on cement production.

## Discussion

### Greenhouse gases and the global warming

When the literature was analyzed, vast amounts of environmental pollutants, encompassing SO_2_, NO_*x*_, CO, and PM, were discharged during cement production (Lei et al. 2011). The production of cement involves the high-temperature calcination of carbonate minerals, resulting in clinker formation and the release of CO_2_ into the atmosphere (Xi et al. 2016). CO_2_ emissions from cement production stem from two primary sources. Firstly, a chemical reaction occurs during the production of the central cement component. The cement generates oxides (lime, CaO), and CO_2_ is emitted due to heat effects. These “process” emissions contribute to approximately 5% of total anthropogenic CO_2_ emissions, excluding land-use changes (Boden et al. 1999). Secondly, nonrenewable energy combustion is used to heat raw materials to temperatures exceeding 1000 °C (IEA 2022b). Around 90% of worldwide CO_2_ emissions from industrial processes result from cement-related activities (M. E. Bildirici 2019, 2020) and the cement industry’s combined emissions account for approximately 8% of the global CO_2_ output (Andrew 2018; Le Quéré et al. 2018).

Overall, a variety of gases, called greenhouse gases (GHG), contribute to the greenhouse effect and global warming. The sizable portion of GHG emissions is dominated by CO_2_ emissions (US EPA 2023). US Environmental Protection Agency reports that the shares of GHGs are CO_2_ at 79.4%, methane (CH4) at 11.5%, nitrous oxide at 6.2%, and the remaining GHGs are fluorinated gases with a total contribution of 3% to the GHG effect (US EPA 2023). As the main driver of climate change due to the GHG effect, the recent positive trend of CO_2_ in the last century is considered a result of human activity due to the burning of fossil fuels (coal, oil, and natural gas), deforestation, and industrial processes, and about more than 65% reduction of current CO_2_ releases is needed to achieve environmental goals (Belbute & Pereira 2020). Therefore, CO_2_ is among the top contributors to global climate change and the reversal necessitates great political commitment.

### Historical relations among cement production, cement-induced CO_2_ emissions, and business cycles

As confirmed by our empirical findings, cement production and CO_2_ emissions are interrelated. Cement production is a derived demand of construction investments accelerating as economic growth accelerates. It is shown that the cement and construction industry and economic growth relation have important linkages (M. E. Bildirici 2019). Economic production is known to follow fluctuations known as business cycles, which include expansionary and recessionary periods. Throughout history, other factors that led to abrupt changes in the production cycle include economic crises, the Great Depression in 1929, the 1973 Oil Crisis, and World Wars (WWI and II). Not only do economic cycles have strong ties with cement as an important ingredient for construction, but also cement industry is also expected to follow a similar pattern possessing expansionary and contractionary episodes in line with the business cycle. Therefore, business cycles also create a derived cement demand during expansions, after crises and recessions. Hence, acceleration in cement production following recessions and even during recessions due to policies encouraging economic growth to overcome such downturns. Such cyclical behaviors in economic business cycles are nonlinear and due to their relation with economic activity, cement production also follows cycles and nonlinearity. Further, cement production is an important emitter of CO_2_. The overlook suggests that the production process of cement and the demand for energy that cement production necessitates are among the top channels that lead to environmental degradation. Various factors with relations to cement production include urbanization and the land-use change (Mishra et al. 2022; Zhou et al. 2021), which contribute to CO_2_ emissions.

**Fig. 1:**
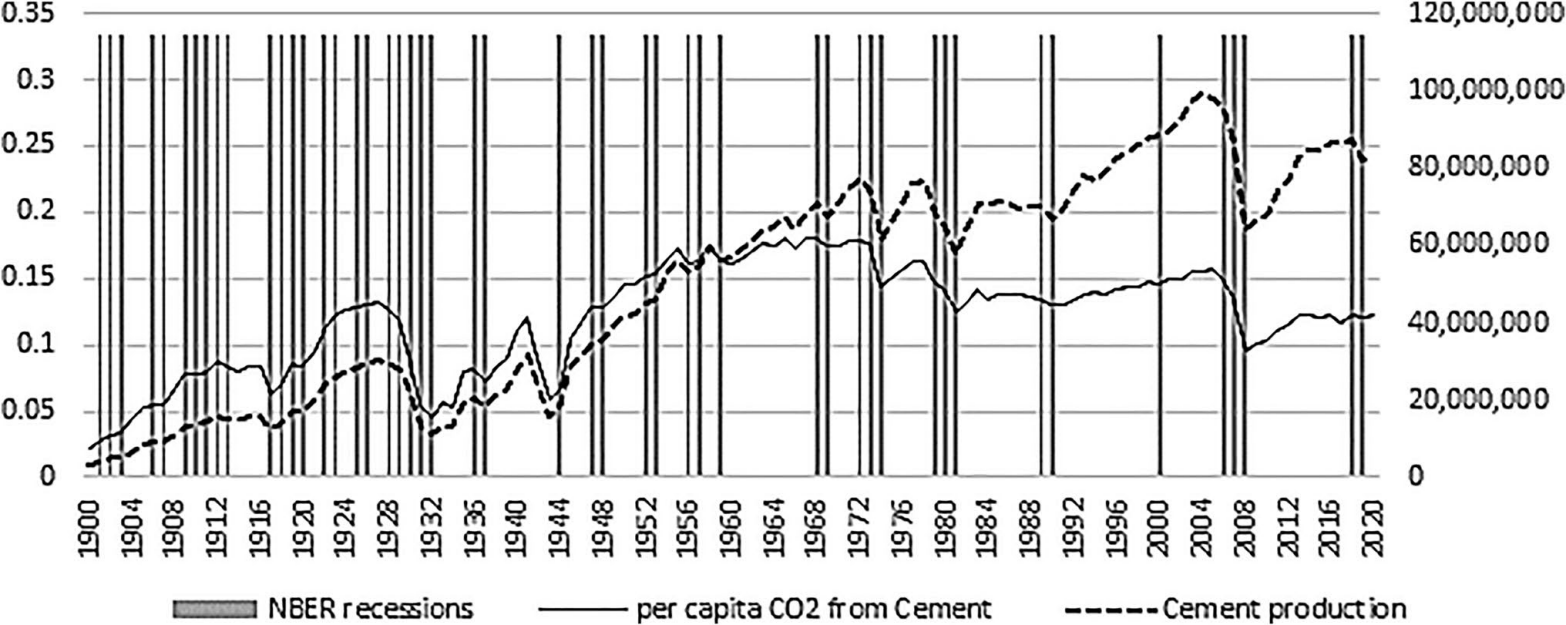
NBER recessions, cement production, and CO_2_ emissions from cement production in the USA, 1900–2021. *Source*: U.S. Geological Survey and NBER. Note that cement production (right-hand side) is in billion metric tons. CO_2_ emission from cement is for tons of CO_2_ from 1 ton of cement produced and is per capita

Yearly cement production (solid line) and CO_2_ emissions from cement production (dashed line) are depicted in Fig. 1 for the 1900–2021 period. The figure also included the recession dates (as a grey bar) obtained from the National Bureau of Economic Recessions (NBER). As seen in Fig. 1, the fluctuations in CO_2_ from cement and cement production are closely linked and a positive association exists between the two series. The inclines and declines in both coincide in terms of occurrence and year and terms of the length of duration in the majority of cases. The recessions in economic business cycles lead to declined economic production, coupled with both declines in CO_2_ emissions from cement and cement production in the USA. This relation becomes clearer, especially during the 1929 Great Depression and 2008 Great Recession with sharp declines in both series. The declines also coincide with the NBER dating of recessions. Another example is the decline in CO_2_ emissions and cement production during the first year of COVID-19 in 2020, which was reversed afterward during the economic expansion that followed in 2021. Hence, the fluctuations in cement production are argued to be in line with economic business cycles, consisting of expansionary and recessionary episodes, leading to similar cycles in cement production and cement-based CO_2_ emissions.

However, there are exceptions such as disputes and wars. During these periods, cement production could increase leading to increased CO_2_. Historical experience shows that during and after periods of conflicts, wars, and economic crises, economic construction investments accelerate. Overlook is construction and cement are cyclical and subject to nonlinearity and so are the emissions. Another point is that expansions are relatively longer lasting compared to recessions. Such economic growth periods lead to inclined cement production and CO_2_ emissions. It is convenient to accept that economic recessions and crises are periods during which the policymakers aim to encourage the economy with expansionary economic policies. During such policies, construction and cement production is an important sector to achieve economic growth. Last but not least, depending on the stage of the economy, the long-run relationship between cement consumption and CO_2_ emissions is also bound by the state of the economy.

History also shows that wars generate demand for construction during the process and the reconstruction period afterward. The statistics for the last century confirm that global cement production has amplified more than 30 times when economic growth accelerated in the 1950s following World War II (WWII) and the demand for cement production fast-tracked due to urban reconstruction of the after-war Europe and participant countries of WWII (Diefendorf 1989). Further, during the expansionary period after the 1980s and 1990s, cement consumption achieved a second period of upward trend, an increase of cement production nearly 4 times in the post-1980s and 1990s, and yearly cement production reached 0.5 tons per person in the world in mid-2010 (Andrew 2018). The post-1980s period corresponds to trade liberalization and globalization policies in the world. Along with post-conflict periods, cement production accelerates after economic recessions and after economic crises. The prominent crises include the Great Depression in 1929, the oil crises and their aftereffects after 1973, the exchange rate mechanism (ERM) in the late 2000s, the Southeast Asia crisis in the mid-1990s, and the Great Recession in 2008, which followed economic policies aimed at acceleration of economic growth and CO_2_ (M. Bildirici & Ersin 2018b). More recently, following the COVID-19 pandemic, nations also applied economic growth policies economic sudden-stop in early 2020. Following lock-downs the end of 2020 and year 2021 experienced high inclines in greenhouse gases and the recent economic recovery from COVID-19 in 2021 is a “carbon-intensive recovery” with a more than 1200-Mt increase in CO_2_ releases in a single year (M. Bildirici & Ersin 2018b). This incline in emissions has been drastically more than those observed during the recovery periods following the 2009 financial crisis (M. Bildirici & Ersin 2018b). As a result, the econometric models focusing on cement and CO_2_ emissions require the integration of nonlinear dynamics taken expansionary and recessionary regimes in the long-run relations.

**Fig. 2:**
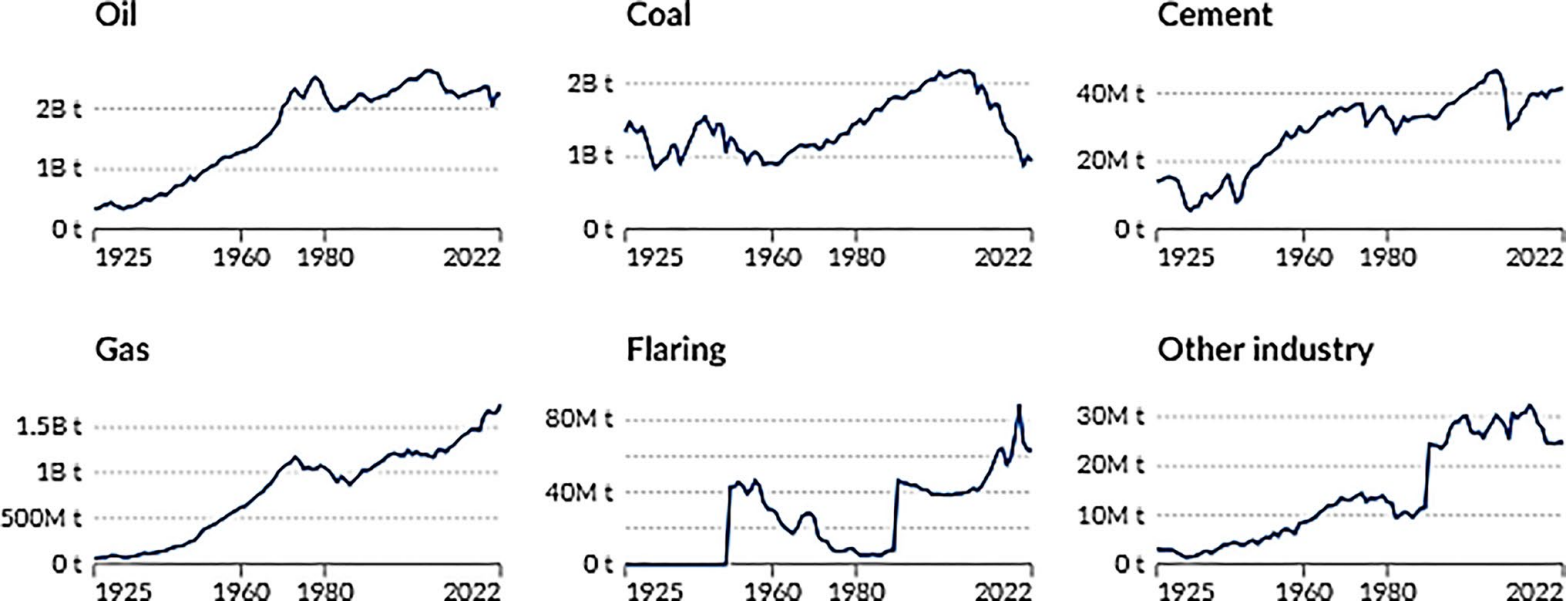
CO_2_ emissions in the USA resulting from cement and other industries and CO_2_ emissions produced by different energy sources, 1920–2022. *Source*: Global Carbon Budget

As shown in the empirical section, the cement-induced CO_2_ emission fluctuations are in close synchronization with economic business cycles. Furthermore, the cement industry’s CO_2_ emissions are closely related to the source of energy. Figure 2 depicts the CO_2_ emissions resulting from cement and other industries in addition to the CO_2_ emissions from different sources of energy, specifically focusing on the fossil-fuel energy types including oil, coal, gas, and flaring in the USA for the 1920–2022 period. The overlook suggests an upward trend of CO_2_ emissions from cement historically, similar to the upward trend followed by CO_2_ emissions from different energy sources. For the whole period, oil is a major emitter with a nonreversing upward trend. After WWII, both oil- and coal-based CO_2_ emissions continued to incline similar to cement-induced CO_2_ emissions. While the pace of oil- and coal-based CO_2_ slowed down, especially for coal after the 2000s, gas and flaring became the major emitters of CO_2_ as the economic development and the necessary energy inclined. The slowing pace of the upward trend of oil- and coal-based CO_2_ is coupled with cement-induced CO_2_. Among all CO_2_ sources, Fig. 2 also depicts the sharp fluctuations in the 1929 Great Depression, 1973 Oil Crisis, and 2008–2009 Global Crisis.

**Fig. 3:**
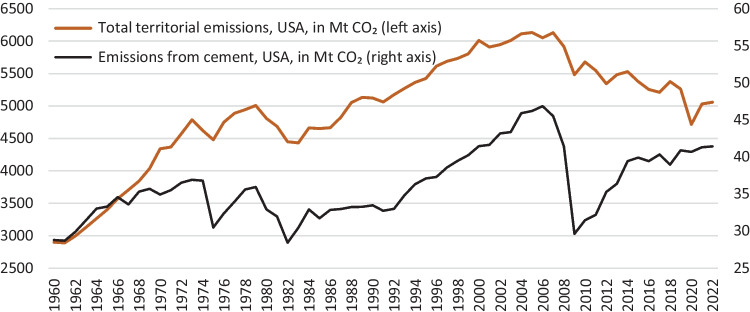
CO_2_ emissions (given in orange, values on the left axis) in the territorial USA, and CO_2_ emissions specifically from cement production (black, right axis), in Mt CO_2_, 1960–2022. *Source*: Global Carbon Budget

Figure 3 aims to provide a focused look at the comparison between total territorial CO_2_ emissions and cement-induced CO_2_ emissions in the USA. The overlook highlights the close ties between the CO_2_ emissions of the economy and the CO_2_ emissions from the cement industry. The total territorial CO_2_ emissions (in orange, values on the left axis) followed an upward trend towards the 1970s, which is closely followed by the cement-induced CO_2_ emissions. The period ended with the transformation of industrial production by reducing the dependence on oil, which occurred following the 1973 Oil Crisis in the USA. The total CO_2_ emissions gained back its rally in the early 1980s for almost 3 decades without a significant interruption, except for minor slow-downs during economic recessions, which are quite negligible. The same pattern is followed by the cement-induced CO_2_. The rally of CO_2_ emissions ended after the economic boom in 2007 and after the 2009 crisis after which the USA started to adopt policies to control the enormous level of CO_2_ emissions. Though the climb is reversed, the amounts should be carefully addressed. The CO_2_ emissions in 2022 were 5000 Mt a year, equal to the amount in the early 1970s when the economy was highly oil-dependent in production and energy. For cement-induced CO_2_ emissions, the level reached is far worse; it is close to 45 Mt of CO_2_, far above the levels in the 1970s. CO_2_ emissions from cement production are given with the black line in Mt CO_2_.

The data in Fig. 3 focuses specifically on the CO_2_ produced by the production processes and avoids the energy consumption in the industry. Combined with it, the total effect depends on the type of energy consumed, and the relative dependence on nonrenewable energy sources is quite high in the USA. The overall result is that the fluctuations in total CO_2_ emissions in the USA and the fluctuations in the cement-induced CO_2_ emissions from the cement industry are highly synchronized, with a strong positive correlation (rho = 0.76). The fluctuations in cement-induced CO_2_ are more pronounced; they closely capture the recessions and crises with significant volatility. Years 1974 and 1981 as well as 2008 denote the most drastic drops in CO_2_ from cement production after a significant reduction in economic production, corresponding to deep economic crises and dispute periods; however, it gained its pace back again in emitting. The examination of Figs. 2 and 3 together confirms the empirical findings of the study and the conclusions. The overall impact of cement production on CO_2_ emissions is positive and is regime-dependent for its positive effect on worsening environmental pollution.

### Cement production as a driver of economic growth

The overall results in this study also confirm the cement industry’s role in driving economic growth indirectly since the economic expansion periods coincide with expansionary periods in cement production. This study does not directly investigate cement production and economic growth relation empirically and for such treatment, readers are referred to M. E. Bildirici (2019, 2020). However, our study points to the industry’s CO_2_ emissions and their relation to cement production cycles characterized by expansionary and contractionary regimes. The findings have important implications. Given the environmental repercussions of the cement sector, our study determines that it is imperative to devise effective environmental remedies and the cement industry should be in the focus not only in terms of its connection to CO_2_ and GHG effect but also in terms of its strong ties with economic growth and business cycles. Hence, the results confirm the necessity to invest in greening cement production technology and to increase energy efficiency in addition to production techniques in cement.

### Policy recommendations

We provided a set of policy aspects in the previous section, which include improving energy efficiency, renewable energy investments, and taking feedback effects in control, especially in relatively higher cement production regimes. Therefore, policies should focus on the reduction of emissions in the sector more with new techniques in cement production, especially during such periods. An interconnected approach is needed that concentrates both on economic and green cement production aspects (Poudyal & Adhikari 2021). A potential reduction in cement production might correlate with slowed economic growth, which is an undesired option for economic policymakers. Therefore, though noting such effects, the reduction of emissions of cement through technology requires immediate action. For such technologies, a set of recent research suggests various methods to reduce the environmental effects of cement production. These include negative emission technologies and decarbonization of the industry (Ren et al. 2023), carbon capture and storage, and nanomaterials and supplementary materials to be used as cement complementary in cement production (Poudyal & Adhikari 2021). The effects of renewable energy and urbanization (Danish et al. 2020) as well as innovation focusing on reducing sectoral emissions in construction have been shown in the literature (Erdoğan et al. 2020). In the context of cement production, policies focusing on sustainable urbanization and clean energy could contribute to the reduction of the indirect emissions led by the cement industry.

For the cement industry, one of the remedies lies in transitioning towards renewable energy sources to replace fossil fuels for cement manufacturing and the high amounts of energy consumption during the production process. To facilitate this transition, policymakers should establish a subsidy program incentivizing companies to adopt renewable energy technologies. Another policy recommendation is to accelerate investments and research and development for energy efficiency in the sector. In a country-wide analysis, energy efficiency surges are shown to have stronger positive effects on the environment compared to renewables. However, it should be kept in mind that there are different forms of renewable energy sources with varying effects on the environment. Further, it is shown that the transition to renewable energy is costly and benefits could be achieved only in the long run (M. Bildirici & Ersin 2023). Policies should re-evaluate the thresholds to achieve and the timeline for net zero carbon transition. This requires steps to be taken to reduce the significant amount of CO_2_ emissions of the industry faster than it is planned in the USA. Lastly, governments addressing environmental issues can achieve preventative health benefits by averting certain illnesses by reducing GHG emissions; the results indicate the need for focusing on the role of industries, and among these, the cement industry is the top third polluter in addition to its production techniques that require a significant amount of energy. To reverse adverse health effects in addition to considering the ties of economic growth and cement production, the cement-induced CO_2_ emissions led to a significant amount of CO_2_ emissions, though some steps are taken in the production techniques of cement compared to the pre-1950s; however, more efforts should be made on production technologies. Such focus can contribute to the formulation of strategies for proactive public health measures, which should include the cement industry, its relation to environmental sustainability economic growth, and energy in the context of the environment-health nexus.

## Conclusion

The cement production activities directly cause emissions during the cement production activities and the size of emissions of such direct emissions deserve special attention for achieving sustainable environment and economic development. The investigation of the long-run effects of cement production is crucial for global warming and climate change. Given the nonlinear nature of the CO_2_ emissions and cement production datasets, the paper aimed at providing a hybrid approach that integrated the Markov-switching models to the ARDL-type cointegration methodology to achieve methods to provide tools to examine the nonlinear and regime-dependent long-run and short-run effects in addition to nonlinear causality modeling. By utilizing a historically long period, covering the 1900–2021 sample, the cement production and its effects on greenhouse gas emissions were examined for the USA. The investigation of the relationship is of crucial importance for sustainability in economic development and the environment since the level of hazardous emissions in the cement production industry is among the top third polluters.

In this study, by employing the Markov-switching to the ARDL analysis, the regression space is divided into two distinct regimes. Regime 1 represents periods of deep recessions and crises, while regime 2 characterizes expansionary periods in the industry. The latter has historically lasted relatively longer in terms of duration. The empirical findings show that cement production has significant positive effects both in the short run and in the long run in both regimes. Further, the regime dating provided by the MS-ARDL model yields insightful findings. The years classified under the crisis regime closely align with severe economic recessions and crises as well as wards such as the 1973 Oil Crisis and World Wars I and II. Regime 2 is notable for encompassing events such as the 1929 Great Depression, the 1973 Oil Crisis, the 2009 Great Recession, and more recently, the economic shutdown resulting from the COVID-19 pandemic. During such periods, especially after the crises, economic policies aim to revive the economic growth back to its track, and the cement industry, being closely tied to economic cycles, demonstrates resilience in short-lived crises. It is observed that cement production persists during shorter recessions, with production continuing and sector inventories being maintained unless the recession is severe and prolonged. Furthermore, before exiting recessionary periods, both cement production and associated CO_2_ emissions from cement production revert to an increasing trend at a faster pace than the previous. Consequently, cement-induced CO_2_ emissions do not decelerate at all in both regimes and emissions continue to escalate as long as cement production activities persist.

Following the generalization of the ARDL model to the novel MS-ARDL, the model is further extended to the MS-VARDL model that capture nonlinear causality relations and the directions of the causal links among the variables analyzed. The novel MS-VARDL method in this study incorporates regime-dependent causal relationships, testing regime-dependent causal directions, which are vital for the determination of the causality direction between cement production and CO_2_ emissions for policy formation. Therefore, the nonlinear causality analyses developed in this study overcome inefficient causal relations in tests that ignore the regime-dependent and nonlinear dynamics in the analyzed series. Further, avoiding such aspects of data would result in incorrectly determined causality directions, leading to inefficient policy recommendations. Hence, the testing of causality with the novel method is of paramount importance, especially for the environment-related emissions dataset and the cement production series subject to nonlinearity in this study.

The regime-specific causality results from the MS-VARDL approach revealed positive and causal effects of cement production in both regimes though the magnitude varies depending on the regime. The findings align with the findings we obtained from the single-regime (i.e., linear) VAR-type causality analysis in terms of capturing the direction of causality in a general sense. However, the regime distinction made in causality testing revealed significant information over the traditional method. The magnitude of the causal effect is notably amplified in the crisis regime, while maintaining causality also in the expansion regime, but with a lower positive effect. Additionally, the methodology employed in the study identified bi-directional causal effects between cement production and CO_2_ emissions, particularly evident in the second regime, suggesting feedback effects between emissions and cement production.

The determination of regime-specific causal effects provided important policy suggestions for the policymakers focusing on the cement industry and its environmental impacts. Accordingly, regime dependence on the causal links necessitates regime-specific policy measures and if the policymaker utilizes traditional approaches, the magnitude of the industry is estimated to be relatively lower than it is in reality. It should also be noted that the USA has a strong commitment to net-zero policies specifically designed for the cement industry towards lowering emissions to net-zero in 2050. However, the findings in our study highlight the underestimation of the severity of greenhouse gas emissions with traditional methods. Given this fact, policy recommendations of the paper underline the necessity of stronger measures towards net-zero policies, state-level government subsidies to achieve greater commitment to renewable energies share in total energy, subsidies to direct investments in the carbon-capture industry, subsidies to the cement industry to achieve pace in energy efficiency, and giving incentives to internalize emission externalities by the industry. Further, as listed in the policy recommendation section, a set of technological improvements are needed to revise the ongoing GHG effects of the cement industry to achieve a sustainable environment and sustainable economic development. Increasing the speed of energy transition towards renewable energy, and reduction of energy consumption with more energy-efficient solutions in the sector, coupled with net-zero policies and technological investments for supplementary material use in the cement industry are among the measures to be taken.

The feedback relation that produces cycles of GHG emissions, especially in the second regime deserves attention. The strong positive ties with economic cycles and industrial production of the industry generate significant amounts of CO_2_ emissions, especially during such periods. The reduction of economic growth is one option but it is not a desired one. Conversely, to cover environmental costs, investments in various emission reduction technologies such as carbon capture and storage are among the viable options that should be taken into focus by policymakers.

The study has limitations due to the availability of data. The nonlinear method utilized in this study requires a long span of data and a large sample size; hence, the study’s empirical focus is restricted to the USA and the 1900–2021 period. As a result, China and India were not examined. For future studies, we suggest extending the analysis to a larger set of countries. Another suggestion is to examine the nexus with a panel of countries.

## Data Availability

Data used in this study are publicly available from the quoted databases reported under the data subheading given in the empirical section. Data are also available upon request from the corresponding author.

## Footnotes

1 Bildirici and Ersin (2023) investigate a set of countries who engage on Industry 4.0 with highest shares of Industry 4.0 innovations in addition to large shares of international trade and the share of fossil-fuel energy use in these countries are interestingly high, 82% in the USA, 87.67% in China, 93.03% in Japan, 78% in Germany, 74% in Canada, and 80% in UK. Lowest practice is achieved by France with 46% of fossil-fuel energy use in total.

2 China leads global cement production by a significant margin, estimated at 2.1 billion metric tons in 2022, representing over half of the world’s total cement output. India follows as the second-largest cement producer globally, with production totaling 370 million metric tons in 2022. Vietnam ranked third in global cement production for that year, producing 120 million metric tons (source: https://www.statista.com/statistics/1087115/global-cement-production-volume/).

3 Such innovations will provide important insights to reduce the emission effects of cement production. However, due to the focus of the study, this literature is kept out of focus in the study.

4 The USA, which produces cement throughout 37 states, is the third-largest cement manufacturer in the world as of 2000. In addition to being a major source of industrial process-related emissions in the USA, cement production also contributes significantly to CO_2_ emissions not only from processing, but also from combustion. The process necessitates high temperatures which is achieved largely with 12% fuel burning, 72% with coal energy, and the rest with various sources including natural gas, coke, and oil (Hanle et al. 2004).

5 The empirical findings revealed the nonlinear association between cement production, air pollution, economic growth, and mortality rate with Bayesian MS-VAR and MS-Bayesian Granger causality tests for the USA, Turkey, India, Brazil, and China and findings confirmed cement production as Granger causes not only air pollution and economic growth, but also mortality rates in all regimes for all countries analyzed (M. E. Bildirici 2020).

6 Following these critiques regarding the efficiency of the test, McNown et al. (2018) proposed a bootstrapped ARDL (BARDL) test as an accompanying test to the *F*_*PSS*_ test and the bound critical values of Pesaran et al. (2001). In the proposed BARDL method (McNown et al. 2018), the utilization of three tests are suggested as compulsory to determine the presence of no-cointegration, cointegration, and degenerate cases. In the state of degenerate-2, the supplied bound critical values of Pesaran et al. (2001) are inefficient and under degenerate case-1, the method is effective.

7 The STARDL method relaxes the form of nonlinearity by allowing nonlinearity both in the short-run and long-run dynamics and derive the relevant tests. For the STARDL modeling and relevant nonlinear cointegration testing, readers are referred to M. Bildirici and Ersin (2018b). The model generalizes STAR models of Granger and Teräsvirta (1993) and Luukkonen et al. (1988) to nonlinear ARDL.

8 Further, STAR type nonlinear models allow capturing different speed of transition between regimes, both smooth and abrupt, However, MS models and threshold models are efficient under abrupt and sudden regime shifts.

9 The MS-VEC model is applied to environmental degradation, hydropower, and nuclear energies for their asymmetric long- and short-run relations (Bildirici 2020).

10 Note that the representation above is a theoretical representation and Davies type linearity tests to determine the number of regimes generally selects 2 regimes and for selected set of applications, 3 regimes.

11 These techniques, in contrast to conventional vector autoregressive (VAR) and vector error correction (VEC) models, also made it possible to evaluate the model without using dummy variables and to examine various stages of the economy.

12 The model presented is a Markov-Switching Intercept and Heteroskedasticity (MSIH-VEC), no regime switching allowed in the long-run and short-run parameters. However, due to the regime switching being introduced to the drift term, the filtered residual is already affected from the changes in drift.

13 The pioneering papers that provide a historical perspective (Grossman & Krueger 1991; Stern et al. 1996) are generalized to nonlinearity with a set of studies. Studies suggest a coverage of centuries long data to examine nonlinear relations among and cycles of emission and GDP growth rates historically (M. Bildirici & Ersin 2018b; Ersin 2016).

14 1954 and 1961 have 10 months lasted recessions; 1958, 1991, and 2001 recessions lasted 8 months; 1970 recession lasted 11 months. After 1970, 1974 recession lasted 16 months, relatively a long-lasting recession which followed the oil crisis. A deep recession occurred between 1975 and mid 1976. In early 1980s, policies included trade liberalization policies in the world which had implications as a short recession that did not affect the cement industry. A difference in the table is 1982–1983 recession which lasted 16 months. However, the model did not capture a contraction in the period. If data investigated, we noted no drastic decline in the cement production for this period, providing a special case.

15 In addition, following the proposal given in the “Methodology” section, we calculated *F* MS-ARDL statistics for single regimes only. Given that the model has 2 variables and 2 states, after estimating an MS(2)-VARDL(2) model, 4 hypotheses are tested by applying zero restrictions. At 5% significance level, results suggest that if emission variable is the dependent variable, cointegration cannot be rejected for regime 1 and regime 2 in addition to nonlinear cointegration in both regimes. In contrast, if cement production is taken as the dependent variable, there exists no cointegration in regime 1, inconclusive result in regime 2, and no cointegration in both regimes, i.e., no nonlinear cointegration.

16 A model with 3 regimes is also estimated; however, the estimation led to inconsistent results. Though the sample covers a long period of 1900–2021, the dataset is yearly and sample size restricts estimation of nonlinear models with more than 2 regimes. Therefore, two regimes are assumed.

17 To save space, the MS-VARDL estimation results are reported. They are available upon request. In the determination of signs of causality, the statistical significance of the parameters, sign, and size of parameter estimates are crucial.

## References

[CR1] Alkama R, Cescatti A (2016) Climate change: biophysical climate impacts of recent changes in global forest cover. Science 351(6273):600-604. 10.1126/SCIENCE.AAC8083 26912702 10.1126/science.aac8083

[CR2] Andrew RM (2018) Global CO_2_ emissions from cement production. Earth Syst Sci Data 10:195-217. 10.5194/essd-10-195-2018

[CR3] Andrew RM (2022) Global CO_2_ emissions from cement production. Zenodo. https://zenodo.org/records/7081360#.Y8GagnZBy5d. Accessed 1 May 2024

[CR4] Anjum Z, Burke P, Gerlagh R, Stern D, Anjum Z, Burke P, Gerlagh R, Stern D (2014) Modeling the emissions-income relationship using long-run growth rates. Centre Clim Energy Policy, Crawford Sch Public Policy, Australian Natl Univ. 10.22004/ag.econ.249422

[CR5] Banerjee P, Arčabić V, Lee H (2017) Fourier ADL cointegration test to approximate smooth breaks with new evidence from Crude Oil Market. Econ Model 67:114-124. 10.1016/J.ECONMOD.2016.11.004

[CR6] Bekun FV, Alola AA, Gyamfi BA, Kwakwa PA, Uzuner G (2022) Econometrics analysis on cement production and environmental quality in European Union countries. Int J Environ Sci Technol. 10.1007/s13762-022-04302-9

[CR7] Belbute JM, Pereira AM (2020) Reference forecasts for CO2 emissions from fossil-fuel combustion and cement production in Portugal. Energy Policy 144:111642. 10.1016/J.ENPOL.2020.111642 32565609 10.1016/j.enpol.2020.111642 PMC7295499

[CR8] Bildirici ME (2019) Cement production, environmental pollution, and economic growth: evidence from China and USA. Clean Technol Environ Policy 21(4):783-793. 10.1007/s10098-019-01667-3

[CR9] Bildirici ME (2020) The relationship between cement production, mortality rate, air quality, and economic growth for China, India, Brazil, Turkey, and the USA: MScBVAR and MScBGC analysis. Environ Sci Pollut Res 27:2248-2263. 10.1007/s11356-019-06586-w 10.1007/s11356-019-06586-w 31776902

[CR10] Bildirici M, Ersin Ö (2018a) Markov-switching vector autoregressive neural networks and sensitivity analysis of environment, economic growth and petrol prices. Environ Sci Pollut Res 25(31):31630-31655. 10.1007/s11356-018-3062-3 10.1007/s11356-018-3062-3 30206834

[CR11] Bildirici M, Ersin ÖÖ (2018b) Economic growth and CO2 emissions: an investigation with smooth transition autoregressive distributed lag models for the 1800–2014 period in the USA. Environ Sci Pollut Res 25(1):200-219. 10.1007/s11356-017-0244-3 10.1007/s11356-017-0244-3 28983717

[CR12] Bildirici M, Ersin ÖÖ (2023) Nexus between Industry 4.0 and environmental sustainability: a Fourier panel bootstrap cointegration and causality analysis. J Clean Prod 386(135786):1-18. 10.1016/J.JCLEPRO.2022.135786

[CR13] Boden TA, Marland G, Andres RJ (1999) Global, regional, and national fossil-fuel CO2 emissions (1751–2014) (V. 2017). 10.3334/CDIAC/00001_V2017

[CR14] Broock WA, Scheinkman JA, Dechert WD, LeBaron B (1996) A test for independence based on the correlation dimension. Economet Rev 15(3):197-235. 10.1080/07474939608800353

[CR15] Cai B, Wang J, He J Geng Y (2016) Evaluating CO2 emission performance in China’s cement industry: an enterprise perspective. Appl Energy 166:191-200. 10.1016/j.apenergy.2015.11.006

[CR16] Cao Y, Guo L, Qu Y (2022) Evaluating the dynamic effects of mitigation instruments on CO2 emissions in China’s nonferrous metal industry: a vector autoregression analysis. Sci Total Environ 853. 10.1016/j.scitotenv.2022.158409 10.1016/j.scitotenv.2022.158409 36055487

[CR17] Chen W, Yan S (2022) The decoupling relationship between CO2 emissions and economic growth in the Chinese mining industry under the context of carbon neutrality. J Clean Prod 379. 10.1016/j.jclepro.2022.134692

[CR18] Clements MP, Krolzig HM (2002) Can oil shocks explain asymmetries in the US Business Cycle? Advances in Markov-Switching Models 41–60. 10.1007/978-3-642-51182-0_3

[CR19] Costa FN, Ribeiro DV (2020) Reduction in CO2 emissions during production of cement, with partial replacement of traditional raw materials by civil construction waste (CCW). J Clean Prod 276. 10.1016/J.JCLEPRO.2020.123302

[CR20] Danish, Ulucak R, Khan SUD (2020) Determinants of the ecological footprint: role of renewable energy, natural resources, and urbanization. Sustain Cities Soc 54:101996. 10.1016/J.SCS.2019.101996

[CR21] DATIS (2020) Worldwide cement production from 2015 to 2019. Datis export group. https://datis-inc.com/blog/worldwide-cement-production-from-2015-to-2019/#United_States. Accessed 1 May 2024

[CR22] Diefendorf JM (1989) Urban reconstruction in Europe after World War II. Urban Studies 26(1):128-143

[CR23] Dirik C, Şahin S, Engin P (2019) Environmental efficiency evaluation of Turkish cement industry: an application of data envelopment analysis. Energ Effi 12(8):2079-2098. 10.1007/S12053-018-9764-Z/TABLES/6

[CR24] Enders W, Lee J (2012) A unit root test using a Fourier series to approximate smooth breaks. Oxford Bull Econ Stat 74(4):574-599. 10.1111/J.1468-0084.2011.00662.X

[CR25] Engle RF, Granger CWJ (1987) Co-integration and error correction: representation, estimation, and testing. Appl Econ 39(3):107-135. 10.2307/1913236

[CR26] EPA (2023) Sources of greenhouse gas emissions and removals. United States Environmental Protection Agency. https://www.epa.gov/ghgemissions/overview-greenhouse-gases. Accessed 17 Jan 2024

[CR27] Erdoğan S, Yıldırım S, Yıldırım DÇ, Gedikli A (2020) The effects of innovation on sectoral carbon emissions: evidence from G20 countries. J Environ Manage 267:110637. 10.1016/J.JENVMAN.2020.110637 32349957 10.1016/j.jenvman.2020.110637

[CR28] Ersin ÖÖ (2016) The nonlinear relationship of environmental degradation and income for the 1870–2011 period in selected developed countries: the dynamic panel-STAR approach. Procedia Econ Finance 38:318-339. 10.1016/S2212-5671(16)30205-2

[CR29] Forzieri G, Alkama R, Miralles DG, Cescatti A (2017) Satellites reveal contrasting responses of regional climate to the widespread greening of Earth. Science 356(6343):1180-1184. 10.1126/SCIENCE.AAL1727 28546316 10.1126/science.aal1727

[CR30] Gao T, Shen L, Shen M, Liu L, Chen F, Gao L (2017) Evolution and projection of CO2 emissions for China’s cement industry from 1980 to 2020. Renew Sustain Energy Rev 74:522-537. 10.1016/j.rser.2017.02.006

[CR31] Granger C, Teräsvirta T (1993) Modelling non-linear economic relationships. Oxford University Press, Oxford

[CR32] Grossman GM, Krueger AB (1991) Environmental impacts of a North American free trade agreement. Natl Bur Econ Res Work Pap Ser 3914(3914):1-57. 10.3386/w3914

[CR33] Hamilton JD (1989) A new approach to the economic analysis of nonstationary time series and the business cycle. Econometrica 57(2):357. 10.2307/1912559

[CR34] Hanle LJ, Jayaraman KR, Smith JS (2004) CO_2_ emissions profile of the U.S. cement industry (working papers). https://www3.epa.gov/ttnchie1/conference/ei13/ghg/hanle.pdf. Accessed 12 Jan 2024

[CR35] Hoffman FM, Randerson JT, Arora VK, Bao Q, Cadule P, Ji D, Jones CD, Kawamiya M, Khatiwala S, Lindsay K, Obata A, Shevliakova E, Six KD, Tjiputra JF, Volodin EM, Wu T (2014) Causes and implications of persistent atmospheric carbon dioxide biases in Earth System Models. J Geophys Res Biogeosci 119(2):141-162. 10.1002/2013JG002381

[CR36] Huang PJ, Huang SL, Marcotullio PJ (2019) Relationships between CO2 emissions and embodied energy in building construction: a historical analysis of Taipei. Build Environ 155:360-375. 10.1016/J.BUILDENV.2019.03.059

[CR37] IEA (2022a) Southeast asia energy outlook 2022: key findings. International energy agency world energy outlook 2022. https://www.iea.org/reports/southeast-asia-energy-outlook-2022. Accessed 1 May 2024

[CR38] IEA (2022b) Renewable energy market update 2022: outlook for 2022 and 2023. International energy agency IEA 50. https://www.iea.org/reports/renewable-energy-market-update-may-2022. Accessed 15 Jan 2024

[CR39] IEA (2022c) World energy outlook 2022 Extended dataset. International energy agency IEA 50. https://www.iea.org/data-and-statistics/data-product/world-energy-outlook-2022-extended-dataset. Accessed 2 Feb 2024

[CR40] Kapetanios V, Shin Y, Snell A (2006) Testing for cointegration in nonlinear smooth transition error correction models. Economet Theor 22(2):279-303. 10.1017/S0266466606060129

[CR41] Karlsson I, Rootzén J, Johnsson F (2020) Reaching net-zero carbon emissions in construction supply chains — analysis of a Swedish road construction project. Renew Sustain Energy Rev 120:109651. 10.1016/J.RSER.2019.109651

[CR42] Karlsson I, Rootzén J, Johnsson F, Erlandsson M (2021) Achieving net-zero carbon emissions in construction supply chains — a multidimensional analysis of residential building systems. Dev Built Environ 8:100059. 10.1016/J.DIBE.2021.100059

[CR43] Ke J, Zheng N, Fridley D, Price L, Zhou N (2012) Potential energy savings and CO2 emissions reduction of China’s cement industry. Energy Policy 45:739-751. 10.1016/J.ENPOL.2012.03.036

[CR44] Krolzig HM, Marcellino M, Mizon GE (2002) A Markov-switching vector equilibrium correction model of the UK labour market. Empir Econ 27(2):233-254. 10.1007/s001810100117

[CR45] Krolzig HM, Toro J (2002) Testing for super-exogeneity in the presence of common deterministic shifts. Annales d’Économie et de Statistique 67/68:41–71. 10.2307/20076342

[CR46] Krolzig HM (1997) The Markov-switching vector autoregressive model. In Lecture Notes in Economics and Mathematical Systems (45):46–28. 10.1007/978-3-642-51684-9_2

[CR47] Kumar Mandal S, Madheswaran S (2010) Environmental efficiency of the Indian cement industry: an interstate analysis. Energy Policy 38(2):1108–1118. 10.1016/j.enpol.2009.10.063

[CR48] Le Quéré C, Andrew RM, Friedlingstein P, Sitch S, Pongratz J, Manning AC, Ivar Korsbakken J, Peters GP, Canadell JG, Jackson RB, Boden TA, Tans PP, Andrews OD, Arora VK, Bakker DCE, Barbero L, Becker M, Betts RA, Bopp L, … Zhu D (2018) Global carbon budget 2017. Earth Syst Sci Data 10(1):405–448. 10.5194/ESSD-10-405-2018

[CR49] Lei Y, Zhang Q, Nielsen C, He K (2011) An inventory of primary air pollutants and CO2 emissions from cement production in China, 1990–2020. Atmos Environ 45(1):147-154. 10.1016/J.ATMOSENV.2010.09.034

[CR50] Li Y, Zuo Z, Cheng Y, Cheng J, Xu D (2023) Towards a decoupling between regional economic growth and CO2 emissions in China’s mining industry: a comprehensive decomposition framework. Resour Policy 80:103271. 10.1016/J.RESOURPOL.2022.103271

[CR51] Liang H, Lin S, Wang J (2022) Impact of technological innovation on carbon emissions in China’s logistics industry: based on the rebound effect. J Clean Prod 377. 10.1016/j.jclepro.2022.134371

[CR52] Lin B, Teng Y (2022) Decoupling of economic and carbon emission linkages: evidence from manufacturing industry chains. J Environ Manag 322. 10.1016/j.jenvman.2022.116081

[CR53] Lin B, Zhang Z (2016) Carbon emissions in China’s cement industry: a sector and policy analysis. Renew Sustain Energy Rev 58:1387-1394. 10.1016/J.RSER.2015.12.348

[CR54] Liu Y, Wang J, Wang X, Wu H, Guo F, Song Y (20220 A study of CO2 emissions in China’s domestic construction industry based on non-competitive input-output. Sustain Prod Consum 32:743-754. 10.1016/j.spc.2022.05.024

[CR55] Liu M, Zhang X, Zhang M, Feng Y, Liu Y, Wen J, Liu L (2021) Influencing factors of carbon emissions in transportation industry based on C–D function and LMDI decomposition model: China as an example. Environ Impact Assess Rev 90. 10.1016/j.eiar.2021.106623

[CR56] Lopez R (1994) The environment as a factor of production: the effects of economic growth and trade liberalization. J Environ Econ Manag 27(2):163-184. 10.1006/jeem.1994.1032

[CR57] Luukkonen R, Saikkonen P, Teräsvirta T (1988) Testing linearity against smooth transition autoregressive models. Biometrika 75(3):491-499

[CR58] Martins MA de B, Crispim A, Ferreira ML, dos Santos IF, Melo M de L NM, Barros RM, Filho GLT (2023) Evaluating the energy consumption and greenhouse gas emissions from managing municipal, construction, and demolition solid waste. Clean Waste Syst 4:100070. 10.1016/J.CLWAS.2022.100070

[CR59] McNown R, Sam CY, Goh SK (2018) Bootstrapping the autoregressive distributed lag test for cointegration. Appl Econ 50(130):1509-1521. 10.1080/00036846.2017.1366643

[CR60] Mishra A, Humpenöder F, Churkina G, Reyer CPO, Beier F, Bodirsky BL, Schellnhuber HJ, Lotze-Campen H, Popp A (2022) Land use change and carbon emissions of a transformation to timber cities. Nat Commun 13(1):1-12. 10.1038/s41467-022-32244-w 36042197 10.1038/s41467-022-32244-w PMC9427734

[CR61] Naqi A, Jang JG (2019) Recent progress in green cement technology utilizing low-carbon emission fuels and raw materials: a review. Sustainability 11(2):537. 10.3390/SU11020537

[CR62] Narayan PK (2014) Reformulating critical values for the bounds F-statistics approach to cointegration: an application to the tourism demand model for Fiji. Monash Univ Dept Econ Discuss Pap 02(04):1-39. 10.4225/03/5938ABDA7B4AB

[CR63] Nelson CR, Piger J, Zivot E (2001) Markov regime switching and unit-root tests. J Bus Econ Stat 19(4):404-415. 10.1198/07350010152596655

[CR64] Oke AE, Aigbavboa CO, Dlamini SA (2017) Carbon emission trading in South African construction industry. Energy Procedia 142:2371-2376. 10.1016/J.EGYPRO.2017.12.169

[CR65] Ozturk I, Acaravci A (2013) The long-run and causal analysis of energy, growth, openness and financial development on carbon emissions in Turkey. Energy Econ 36:262-267. 10.1016/j.eneco.2012.08.025

[CR66] Pakdel A, Ayatollahi H, Sattary S (2021) Embodied energy and CO2 emissions of life cycle assessment (LCA) in the traditional and contemporary Iranian construction systems. J Build Eng 39:102310. 10.1016/J.JOBE.2021.102310

[CR67] Pavlyuk D (2017) On application of regime-switching models for short-term traffic flow forecasting. In: Zamojski W, Mazurkiewicz J, Sugier J, Walkowiak T, Kacprzyk J (eds) Advances in dependability engineering of complex systems. DepCoS-RELCOMEX 2017. Advances in intelligent systems and computing, vol 582. Springer, Cham. pp 340–349. 10.1007/978-3-319-59415-6_33

[CR68] Pesaran HM, Shin Y, Smith RJ (2001) Bounds testing approaches to the analysis of level relationships. J Appl Economet 16:289-326. 10.1002/jae.616

[CR69] Pettersson F, Maddison D, Acar S, Söderholm P (2014) Convergence of carbon dioxide emissions: a review of the literature*. Int Rev Environ Resour Econ 7(2):141-178.

[CR70] Poudyal L, Adhikari K (2021) Environmental sustainability in cement industry: an integrated approach for green and economical cement production. Res Environ Sustain 4. 10.1016/J.RESENV.2021.100024

[CR71] Ren M, Ma T, Fang C, Liu X, Guo C, Zhang S, Zhou Z, Zhu Y, Dai H, Huang C (2023) Negative emission technology is key to decarbonizing China’s cement industry. Appl Energy 329. 10.1016/j.apenergy.2022.120254

[CR72] Richardson AD, Keenan TF, Migliavacca M, Ryu Y, Sonnentag O, Toomey M (2013) Climate change, phenology, and phenological control of vegetation feedbacks to the climate system. Agric for Meteorol 169:156-173. 10.1016/J.AGRFORMET.2012.09.012

[CR73] Rodgers L (2018) Climate change: the massive CO_2_ emitter you may not know about. BBC News. https://www.bbc.com/news/science-environment-46455844. Accessed 1 May 2024

[CR74] Saikkonen P (2005) Stability results for nonlinear error correction models. J Econ 127(1):69-81. 10.1016/j.jeconom.2004.03.001

[CR75] Saikkonen P (2008) Stability of regime switching error correction models under linear cointegration. Economet Theor 24(1):294-318. 10.1017/S0266466608080122

[CR76] Selden TM, Song D (1994) Environmental quality and development: is there a Kuznets curve for air pollution emissions? J Environ Econ Manag 27(2):147-162. 10.1006/JEEM.1994.1031

[CR77] Shin Y, Yu B, Greenwood-Nimmo M (2013) Modelling asymmetric cointegration and dynamic multipliers in a nonlinear ARDL framework. Festschrift Honor Peter Schmidt 44:1-35. 10.1007/978-1-4899-8008-3

[CR78] Stern DI (1994) The rise and fall of the environmental Kuznets curve. J Environ Econ Manag 27:147–167. 10.1016/j.worlddev.2004.03.004

[CR79] Stern DI, Common SM, Barbier BE (1996) Economic growth and environmental degradation: the environmental Kuznets curve and sustainable development. In World Development 24(7) 10.1016/0305-750X(96)00032-0

[CR80] Stern DI, Gerlagh R, Burke PJ (2017) Modeling the emissions–income relationship using long-run growth rates. Environ Dev Econ 1–26. 10.1017/S1355770X17000109

[CR81] Supino S, Malandrino O, Testa M, Sica D (2016) Sustainability in the EU cement industry: the Italian and German experiences. J Clean Prod 112:430-442. 10.1016/J.JCLEPRO.2015.09.022

[CR82] Tan C, Yu X, Guan Y (2022) A technology-driven pathway to net-zero carbon emissions for China’s cement industry. Appl Energy 325. 10.1016/j.apenergy.2022.119804

[CR83] Teller P, Renzoni R, Germain A, Delaisse P, D’Inverno H (2000) Use of LCI for the decision-making of a Belgian cement producer: a common methodology for accounting CO2 emissions related to the cement life cycle. In: 8th LCA Case Studies Symposium SETAC-Europe

[CR84] The Guardian (2019) Concrete: the most destructive material on Earth. The Guardian. https://www.theguardian.com/cities/2019/feb/25/concrete-the-most-destructive-material-on-earth. Accessed 14 Dec 2023

[CR85] Tsay RS (1986) Nonlinearity tests for time series. Biometrika 73(2):461. 10.2307/2336223

[CR86] UNCC (2022) Climate plans remain insufficient: more ambitious action needed now. United Nations climate change. https://unfccc.int/news/climate-plans-remain-insufficient-more-ambitious-action-needed-now. Accessed 1 May 2024

[CR87] USGS (2024) National minerals information center: cement statistics and information. U.S. geological survey. https://www.usgs.gov/centers/national-minerals-information-center. Accessed 8 Mar 2024

[CR88] Vorayos N, Jaitiang T (2020) Energy-environmental performance of Thai’s cement industry. Energy Rep 6:460-466. 10.1016/J.EGYR.2019.11.103

[CR89] Wang Y, Zhu Q, Geng Y (2013) Trajectory and driving factors for GHG emissions in the Chinese cement industry. J Clean Prod 53:252-260. 10.1016/J.JCLEPRO.2013.04.001

[CR90] WPR (2024) Cement production by country 2024. World population review. https://worldpopulationreview.com/country-rankings/cement-production-by-country. Accessed 2 Jan 2024

[CR91] Worrell E, Price L, Martin N, Hendriks C, Meida LO (2001) Carbon dioxide emissions from the global cement industry. Annual Rev of Environ and Res 6. 10.1146/annurev.energy.26.1.303

[CR92] Wu T, Ng ST, Chen J (2022) Deciphering the CO2 emissions and emission intensity of cement sector in China through decomposition analysis. J Clean Prod 352. 10.1016/J.JCLEPRO.2022.131627

[CR93] Xi F, Davis SJ, Ciais P, Crawford-Brown D, Guan D, Pade C, Shi T, Syddall M, Lv J, Ji L, Bing L, Wang J, Wei W, Yang KH, Lagerblad B, Galan I, Andrade C,Y Zhang Y, Liu Z (2016) Substantial global carbon uptake by cement carbonation. Nat Geosci 9(12):880-883. 10.1038/ngeo2840

[CR94] Xia D, Zhang L (2022) Coupling coordination degree between coal production reduction and CO2 emission reduction in coal industry. Energy 258. 10.1016/j.energy.2022.124902

[CR95] Xin D, Ahmad M, Khattak SI (2022) Impact of innovation in climate change mitigation technologies related to chemical industry on carbon dioxide emissions in the United States. J Clean Prod 379. 10.1016/j.jclepro.2022.134746

[CR96] Xu JH, Fleiter T, Eichhammer W, Fan Y (2012) Energy consumption and CO2 emissions in China’s cement industry: a perspective from LMDI decomposition analysis. Energy Policy 50:821-832. 10.1016/J.ENPOL.2012.08.038

[CR97] Xu B, Xu R (2022) Assessing the role of environmental regulations in improving energy efficiency and reducing CO2 emissions: evidence from the logistics industry. Environ Impact Assess Rev 96. 10.1016/j.eiar.2022.106831

[CR98] Xu H, Li Y, Zheng Y, Xu X (2022) Analysis of spatial associations in the energy–carbon emission efficiency of the transportation industry and its influencing factors: evidence from China. Environ Impact Assess Rev 97. 10.1016/j.eiar.2022.106905

[CR99] Yu Y, Yazan DM, Bhochhibhoya S, Volker L (2021) Towards circular economy through industrial symbiosis in the Dutch construction industry: a case of recycled concrete aggregates. J Clean Prod 293. 10.1016/j.jclepro.2021.126083

[CR100] Zhang Y, Zhang Y, Zhu H, Zhou P, Liu S, Lei X, Li Y, Li B, Ning P (2022) Life cycle assessment of pollutants and emission reduction strategies based on the energy structure of the nonferrous metal industry in China. Energy 261. 10.1016/j.energy.2022.125148

[CR101] Zhang J, Shen J, Xu L, Zhang Q (2023) The CO2 emission reduction path towards carbon neutrality in the Chinese steel industry: a review. Environ Impact Assess Rev 99. 10.1016/j.eiar.2022.107017

[CR102] Zhao Q, Gao W, Su Y, Wang T (2022) Carbon emissions trajectory and driving force from the construction industry with a city-scale: a case study of Hangzhou, China. Sustain Cities Soc 104283. 10.1016/j.scs.2022.104283

[CR103] Zheng J, Chen A, Yao J, Ren Y, Zheng W, Lin F, Shi J, Guan A, Wang W (2022) Combination method of multiple molding technologies for reducing energy and carbon emission in the foundry industry. Sustain Mater Technol e00522. 10.1016/j.susmat.2022.e00522

[CR104] Zhou Y, Chen M, Tang Z, Mei Z (2021) Urbanization, land use change, and carbon emissions: quantitative assessments for city-level carbon emissions in Beijing-Tianjin-Hebei region. Sustain Cities Soc 66:102701. 10.1016/J.SCS.2020.102701

